# Creating a Selective Nanobody Against 3-Nitrotyrosine Containing Proteins

**DOI:** 10.3389/fchem.2022.835229

**Published:** 2022-02-21

**Authors:** Elise M. Van Fossen, Sonia Grutzius, Carl E. Ruby, Dan V. Mourich, Chris Cebra, Shay Bracha, P. Andrew Karplus, Richard B. Cooley, Ryan A. Mehl

**Affiliations:** ^1^ Oregon State University, Department of Biochemistry and Biophysics, Agricultural and Life Sciences, Corvallis, OR, United States; ^2^ Oregon State University, Department of Clinical Sciences, College of Veterinary Medicine, Corvallis, OR, United States; ^3^ Department of Small Animal Clinical Sciences (VSCS), Texas A&M College of Veterinary Medicine and Biomedical Sciences, College Station, TX, United States

**Keywords:** genetic code expansion, nanobody, oxidative post translational modification, nitrotyrosine, single domain antibody

## Abstract

A critical step in developing therapeutics for oxidative stress-related pathologies is the ability to determine which specific modified protein species are innocuous by-products of pathology and which are causative agents. To achieve this goal, technologies are needed that can identify, characterize and quantify oxidative post translational modifications (oxPTMs). Nanobodies (Nbs) represent exquisite tools for intracellular tracking of molecules due to their small size, stability and engineerability. Here, we demonstrate that it is possible to develop a selective Nb against an oxPTM protein, with the key advance being the use of genetic code expansion (GCE) to provide an efficient source of the large quantities of high-quality, homogenous and site-specific oxPTM-containing protein needed for the Nb selection process. In this proof-of-concept study, we produce a Nb selective for a 3-nitrotyrosine (nitroTyr) modified form of the 14-3-3 signaling protein with a lesser recognition of nitroTyr in other protein contexts. This advance opens the door to the GCE-facilitated development of other anti-PTM Nbs.

## Introduction

Oxidative post-translational modifications (oxPTMs) are formed by small molecule oxidants reacting with proteins under both normal and oxidative stress conditions and their heterogeneity in terms of the types, locations and the extents of the modification make it challenging to pin-point the effects they exert. Regardless, the study of oxPTMs remains critical as these modifications are often identified in disease pathology and show promise as disease prediction tools (Tomin et al., 2019).

The oxPTM 3-nitrotyrosine (nitroTyr, nY) is produced by the formation of peroxynitrite-derived radicals and their subsequent reaction with tyrosine side chains (Souza et al., 2008). Its accumulation in over 100 distinct proteins is associated with numerous disease pathologies (Pacher et al., 2007). NitroTyr-modified proteins have been shown to cause functional changes that can contribute to disease (Franco et al., 2013; Bartesaghi and Radi 2018; Ferrer-Sueta et al., 2018; Radi, 2018; Porter et al., 2020), but this has been possible in only a few cases, as it is challenging to define which specific nitroTyr protein species are innocuous by-products of pathology and which are causative agents. As such knowledge is required for the development of effective therapeutics for oxidative stress-related pathologies, there is a critical need for tools to identify, characterize and quantify nitroTyr-modified proteins.

One such tool has been anti-nitroTyr antibodies (nitroTyr-Abs), which have enabled immune-localization of nitroTyr proteins (Moller et al., 2019) and nitroTyr-specific proteomic profiling (Herce-Pagliai et al., 1998). Most commonly employed is a nitroTyr-Ab specific to the PTM itself (Beckmann et al., 1994), though protein and site-specific nitroTyr-Abs have also been developed (Khan et al., 2017). Despite the utility of nitroTyr-Abs for the identification and enrichment of nitrated proteins, their large size has prevented their use for tracking proteins in live cells, which would allow nitroTyr-induced changes in client binding and subcellular location to be monitored.

Recently, nanobodies (Nbs)—single-domain antibody fragments derived from the heavy-chain of immunoglobulins of camelids and much smaller than Abs (∼15 kDa vs.–150 kDa)—have been shown to be effective for the intracellular tracking of proteins (Moeglin et al., 2021). Additionally, Nbs are well suited for binding epitopes that are inaccessible to traditional Abs due to their protruding, convex paratopes (Muyldermans 2013; Pardon et al., 2014). The ability to generate nitroTyr-protein specific Nbs would allow the full suite of Nb capabilities to be harnessed for both *in vitro* and in cell work, including for instance, the modulation of protein activity by targeting nitroTyr proteins with a covalent nanobody (or “GlueBody”) for degradation in order to observe the downstream effects on redox signaling (Bery et al., 2019; Cheloha et al., 2020; de Beer and Giepmans 2020; Zhang et al., 2021).

Nb selections require high-quality, homogenous target protein (Pardon et al., 2014), and the inability to make such protein has been a general barrier to creating Nbs against oxPTMs. Traditionally, oxPTMs are chemically introduced into proteins but this process generates multiple types of chemical modifications with minimal ability to control site-specificity (Ischiropoulos et al., 1992; Neumann et al., 2008; Gerding et al., 2019). Indeed, to our knowledge, no Nb has yet been developed against any oxPTM and only a single example has been published for any type of PTM (Moeglin et al., 2021). An avenue to bypass this barrier for PTMs is genetic code expansion (GCE), which can install a variety of PTMs into proteins to generate large quantities of homogenous, site-specific PTM-containing proteins (Neumann et al., 2008; Franco et al., 2013; Cooley et al., 2014a; Cooley et al., 2014b; DiDonato et al., 2014; Franco et al., 2015; Porter and Mehl 2018; Porter et al., 2019; Randall et al., 2019; Beyer et al., 2020; Jang et al., 2020; Porter et al., 2020). In GCE, non-canonical amino acids (ncAAs) are site-specifically incorporated into stop codons *via* orthogonal amino-acyl tRNA synthetase (aaRS)/tRNA_UAG_ pairs. As GCE tools already exist to incorporate nitroTyr, we sought here to use GCE-produced nitroTyr-modified proteins as proof of concept to show how Nbs can be developed against oxPTM targets.

As trial nitrated targets, we selected two hub proteins involved in cell signaling pathways: 14-3-3 (Pennington et al., 2018) and calmodulin [CaM; (Smallwood et al., 2003)]. Both govern critical processes in biology and contain multiple biologically relevant sites of tyrosine nitration (Ghesquiere et al., 2009; Nuriel et al., 2015; Zhao et al., 2017; Porter et al., 2020). After generating a nitroTyr-Nb library from an immunized camelid, we performed selections with the most promising candidate protein target and obtained a single Nb with a reasonable level of selectivity for certain nitrated protein targets, even in highly proteinaceous solutions. Furthermore, as a step toward developing covalent Nb binders, we incorporate a photocrosslinking ncAA at multiple positions in this anti-nitroTyr Nb. Though these positions did not result in high yielding crosslinking, we showcase the versatility of GCE-technology in Nb development, from Nb-selections to Nb engineering.

## Materials and Methods

### Non-Canonical Amino Acids

3-nitro-tyrosine (nitroTyr, nY) and 4-azido-phenylalanine (azidoPhe, pAzF) were purchased from Alfa Aesar (Cat. no. A11018) and Chem impex (Cat. no. 06162). NcAA solutions were prepared fresh before each use by suspending the amino acids in water and solubilizing with 1–2 M equivalents of NaOH for a final concentration of 100 mM ncAA.

### Molecular Cloning

Molecular cloning of plasmids used in this study (Supplementary Table S1) were constructed in the following manner. Previously published sequences containing at least 25 bp of homology at their flanking ends to either the vector backbone or other fragments, were optimized for expression in *E. coli* and synthesized by Integrated DNA Technologies (Coralville, IA, USA, Supplementary Table S2). Gene fragments, as well as fragments containing amber stop codons were amplified with primers listed in Supplementary Table S3 using touchdown PCR (Green and Sambrook 2018) and the resulting products were separated on 0.8–1.2% (w/v) agarose gels and purified using Gene Jet Gel Extraction Kit (ThermoFisher Scientific, Waltham, MA, United States) according to the manufacturer’s instructions. Vector backbones were prepared through restriction digestion and purified through gel extraction. Fragments and vector backbones were then ligated using the SLiCE cloning protocol (Zhang et al., 2012) transformed into chemically-competent DH10B *E. coli* cells and selected on LB agar plates containing the appropriate antibiotic. Colonies were selected and propagated prior to purification. Genetic sequences of each plasmid were confirmed using Sanger sequencing. The primers and template used for PCR amplification, as well as the vector backbone and restriction enzymes used for linearization are summarized for each plasmid in Supplementary Table S1. Plasmids not prepared in this study were prepared previously (see references in Supplementary Table S1).

### Protein Expression

For the expression of nitroTyr and azidoPhe proteins, each TAG site containing expression plasmid was co-transformed with a GCE-machinery plasmid (see Supplementary Table S1) into BL21ai cells. All pDule2 machinery plasmids contain a p15a origin of replication and constitutively express the indicated amber suppressing *Methanocaldococcus jannaschii* (*Mj*) aminoacyl tRNA synthetase (aaRS)/tRNA_CUA_ pair. Cultures (5 ml) were inoculated by scraping a swath of cells containing the appropriate plasmids from a fresh LB agar plate and were grown overnight in 2XYT (no more than 16 h) in the presence of appropriate antibiotics. Overnight cultures were then used to inoculate expression cultures of ZY-AIM media [0.1–1.0 L, (Studier 2005)], supplemented with appropriate antibiotics. For the expression of nitroTyr and azidoPhe proteins, the media was also supplemented with nitroTyr and azidoPhe (at final concentration of 1 mM, diluted from a freshly prepared 100 mM solution as described above). Cultures were grown with constant shaking at 275 rpm in baffled flasks at 37°C until they reached an OD_600_ of 1.5, wherein they were induced with a final concentration of 0.1% arabinose. Upon induction, the temperature was decreased to 25°C and cultures expressed for an additional 30 h prior to cell harvesting.

### Protein Purification

Cell pellets were resuspended in a lysis/wash buffer (50 mM Tris, 500 mM NaCl, 5 mM imidazole, pH 7.5) and lysed using a M-110P microfluidizer system (Microfluidics Corp, Newton, MA, United States) set at 18,000 psi. Cell debris was pelleted at 20,000 rcf for 25 min at 4°C and clarified cell lysate was recovered. To bind His_6_-tagged protein, clarified cell lysate was then incubated with TALON resin (Takara Bio, Japan) at 4°C for 1 h with rocking. Resin was collected and extensively washed with 50 resin bed volumes (bv) of lysis buffer. At this point, for proteins containing an N-terminal His-*bd*NEDD8 tag (Frey and Gorlich 2014), the protein-bound resin was resuspended in 3 bvs of storage buffer (50 mM Tris, 500 mM NaCl, 10% glycerol). His-tag free *bd*NEDP1 (a kind gift from Dirk Gorlich, Addgene #104129, 100 nM) was added to the resuspension and the solution was incubated for 1.5 h at room temperature with rocking. The resin was retained in a column and cleaved nanobody was collected in the flow-through.

For all other constructs, bound protein was eluted from the TALON resin by incubation with five bv’s of Elution Buffer (lysis buffer with 200 mM imidazole) and desalted into storage buffer using a PD-10 desalting column (GE Health Sciences). When cleavage of a His_6_-SUMO tag was required, Ubiquitin-Like Protease 1 (ULP1) was added to the desalted solution and was incubated overnight at 4°C. Following incubation, cleaved protein was re-flowed over TALON resin to bind the cleaved tag, and the flow-through containing the Nb was collected.

Nb-G5 was further purified by size exclusion chromatography using a Superdex 200 10/300 column in storage buffer. When necessary, the protein solution was concentrated by using a 3 kDa MWCO Vivaspin spin-concentration filter (GE Health Sciences). Protein concentration was determined by absorbance at 280 nm using primary sequence calculated extinction coefficients, flash-frozen in liquid nitrogen and stored at −80°C.

### Mass Spectrometry of Nitrated Proteins

Purified protein was exchanged into LC-MS grade water or 50 mM tri-ethyl-ammonium bicarbonate with PD-10 columns (Cytiva) diluted to 50 μM and analyzed with the Waters Synapt G2 Mass Spectrometer at the Mass Spectrometry Facility at Oregon State University. The deconvoluted masses were obtained by using Waters MassLynx MaxEnt1 software.

### Expression and Purification of Biotinylated Proteins for Biolayer Interferometry

Biotinylated 14-3-3 proteins were expressed in BL21ai by co-transforming pBAD-AVI-14-3-3-His plasmids (Supplementary Table S1) with the GCE machinery plasmid pDule2-3-nitroTyr-A7 (Addgene #174079) and the pEVF-GST-BirA plasmid. This plasmid expresses the fusion protein GST-BirA in a vector compatible with GCE machinery plasmids and most common expression plasmids (RSF origin, CmR antibiotic resistance). pBAD-AVI-eGFP-His was also co-transformed with pEVF-GST-BirA to generate biotinylated eGFP as a control for Biolayer Interferometry (BLI) experiments.

Expression of biotinylated 14-3-3ß proceeded as described above except with the addition of 50 µM biotin and 25 µg/μL chloramphenicol to expression cultures, and at an OD of 1.5, 0.1 mM IPTG was added to induce the expression of GST-BirA. The purification of biotinylated 14-3-3ß was identical to non-biotinylated 14-3-3ß and biotinylation of Nb targets was validated by streptavidin motility-shift assay (Fairhead and Howarth 2015).

### Peptide Design for Library Generation

Peptides were designed to encompass nitrated portions of calmodulin (nY99 and nY138) and 14-3-3 (nY133). The peptide for 14-3-3- nY133 BMHI shared a sequence identity of 61.9% with 14-3-3 nitroTyr human isoform b. A peptide for general nitration was also designed, containing multiple nitroTyr flanked by β-sheets. Peptides were designed by following the described parameters for Nb generation against a folded protein (Trier et al., 2012) and were submitted to B-epitope predictors (http://crdd.osdd.net/raghava/abcpred/) to determine epitope effectiveness. The peptides contained a N-terminal cysteine to facilitate keyhole limpet hemocyanin (KLH) conjugation. The peptides were synthesized by Genscript, resuspended in TBS and conjugated to KLH with the Thermo Scientific Imject Maleimide-Activated mcKLH Spin Kit by following the manufacturer’s instructions.

### Nitration of KLH

KLH was nitrated by following a previously described method (Ischiropoulos et al., 1992; Beckmann et al., 1994). Briefly, KLH (100 µg) was diluted into 100 mM potassium phosphate pH 7.4 to a final concentration of 0.160 µg/µL. Peroxynitrite (160 mM stock concentration) was added to the solution to achieve a final concentration of 2 mM. The solution was incubated at room temperature for 14 h, after which nitrated KLH was exchanged into PBS by overnight dialysis at 4°C (MWCO 3,000 Da). Nitration was confirmed by Western Blotting using Millipore’s Polyclonal anti-nitroTyr Ab (Cat # 06-284, see below section “Western Blots”).

### Construction of Nb Display Libraries

#### Immunization of Animal

A healthy Oregon State University owned alpaca was the host for Nb production. The animal was immunized over a series of six injections with a total of 700 µg of a mixture of the nitrated peptides conjugated to KLH in 1X PBS mixed with equal volume (1 ml:1 ml) Sigma adjuvant System. The peptides were injected subcutaneously over the right caudal neck. Pre- and post-immune serum samples were collected. Immunizations were followed by a production bleed not exceeding 200 ml and whole blood collected into heparinized tubes. All animal protocols were approved by the Oregon State University Institutional Animal Care and Use Committee (IACUC).

#### Serum Analysis

Pre- and post-immune serum samples were collected and screened for the presence of Nbs binding nitrated targets. 14-3-3 (WT and nY133), CaM (WT, nY99 and nY138) (5 µg), and HCT116 cell lysate as a positive control were separated on 15% SDS-PAGE gels, transferred to PVDF membrane, blocked with 5% (w/v) nonfat milk in TBST and probed with either pre- or post-immunization serum, rocking for 16 h at room temperature. After rinsing three times with TBST, the membranes were than incubated with anti-llama horseradish peroxidase (HRP) conjugated secondary antibody (goat anti-llama IgG HRP, ab112786, Abcam, Cambridge, United Kingdom) diluted 1:20,000 in 5% nonfat milk/TBST for 1 h at room temperature. The membranes were washed three times with TBST and visualized with Clarity Western ECL Substrate.

#### Library Generation

After the immunization procedure, blood samples were collected (100 ml), peripheral blood lymphocytes (PBLs) were enriched by gradient centrifugation from blood and total RNA was isolated from the PBLs to prepare cDNA (RNAeasy, Qiagen, San Diego, CA, United States). The open reading frames encoding all immunoglobulin heavy-chains were amplified by RT-PCR with primers. Nb open reading frames were amplified through a nested PCR using primers to generate flanking sequences amenable to homologous recombination into pSEX81 (PR3005, Progen Biotechnik GmbH, Heidelberg, Baden- Wuerttemberg, Germany). PCR products (10 µg) were mixed with NcoI/BamHI linearized pSEX81 vector (Progen, 10 µg) and ligated by with T4 DNA ligase and electro-transformed into competent *E. coli* TG1 cells. Transformants were grown in 2TY medium containing 2% glucose and 100 μg/ml ampicillin at 37°C overnight (Sabir et al., 2014).

### Library Selections

The transformed TG1 cells were incubated with hyperphage (PRHYPE, Progen Biotechnik GmbH, Heidelberg, Baden- Wuerttemberg, Germany). The phage particles presenting the VHH library on their tips were collected. Phages containing the Nb fragments were enriched with solid phase panning. As a negative selection, Enzyme-Linked Immunosorbent Assay (ELISA) wells were coated with 5 µg of KLH at 4°C and phage particles were added to the wells and incubated at room temperature for 1.5 h. The unbound phage was moved to another round of negative selection for a total of three rounds. Afterwards, unbound phage were moved to positive selection with nitrated KLH. Bound phages were eluted with 0.1 M triethylamine and used for reinfection of TG1 cells, which were then used for one round of negative selection with WT 14-3-3ß bound by the (6x) His to Pierce^®^ Nickel Coated Plates (ThermoScientific). The unbound phage were used for two subsequent panning rounds of positive sections with nitroTyr 14-3-3ß immobilized to the Ni-coated plates resulting two full length Nb sequences (Nb-G5 and Nb-F110).

### Nb Validation With Dot Blots

Immobilon-FL PVDF Membrane was activated by a brief incubation in methanol then equilibrated in MilliQ water followed by equilibration in Towbin’s buffer [25 mM Tris, 192 mM glycine, 20% (v/v) methanol (pH 8.3)]. Three microlitres of 14-3-3 targets were blotted onto the PVDF membrane obtaining final masses ranging from 0.5 to 5 µg. The membrane was incubated briefly in Ponceau S to visualize the protein load. The membrane was then rinsed with TBST and blocked with 5% milk for 1 hour then rinsed 3 × 5 min with TBST. Nbs were prepared in TBS to achieve a 1:3,000 desired dilution. Nb was incubated with the PVDF membrane at RT at times ranging from 1 h to overnight. After incubation, membranes were rinsed 3 × 5 min with TBST. The secondary antibody, horseradish peroxidase (HRP) conjugated anti-llama, was diluted 1:20,000 in 5% milk and incubated with the PVDF membrane overnight. Bound Nbs were incubated for 1 h at room temperature with an anti-llama HRP conjugated secondary antibody diluted 1:20,000 in 5% nonfat milk/TBST. The membranes were washed three times with TBST and visualized with Clarity Western ECL Substrate.

### Nb Validation With Enzyme-Linked Immunosorbent Assay

Nbs were prepared in TBS to achieve a 1:3,000 desired dilution and were incubated with immobilized WT 14-3-3 (5 µg) and nY 130/133 and nY 213/216 14-3-3 (0.1–5 µg) in Ni-coated ELISA wells. Nbs were incubated at room temperature for 1 hour and was visualized after washing 10x with TBST. Bound Nbs were incubated for 1 h at room temperature with an anti-llama HRP conjugated secondary antibody diluted 1:1,000 in 5% nonfat milk/TBST. The wells were washed ten times with TBST and visualized with Clarity Western ECL Substrate.

### Nb Validation With Biolayer Interferometry

All BLI measurements were made on a fortéBIO (Menlo Park, CA, United States) Octet Red96 system using streptavidin (SA) or nickel (Ni) sensors. Assays were performed in 96-well microplates at 37°C. All sample volumes were 200 µL. SA sensors were loaded with 14-3-3ß homogeneously biotinylated at the N-terminus (0.020 µg/µL). Preliminary assays were done to determine an appropriate amount of biotinylated 14-3-3 to be loaded on the SA sensors, defined as the lowest amount of 14-3-3 that would provide acceptable signal above background for the lowest concentration of Nb-G5 as well as reasonable signal with minimal distortion at near-saturating concentrations of Nb-G5. After loading biotinylated 14-3-3ß onto SA sensors, a baseline was established in buffer composed of 50 mM Tris pH 7.5, 150 mM NaCl and BSA (0.1–1 mg/ml) or Tween (0.05%). Association with the Nb was then carried out in the same buffer for 60–90 s at Nb concentrations that ranged from 0.0125–1.5 μM. Dissociation was subsequently measured the same buffer for 120–300 s. BSA was used in all buffers to reduce non-specific binding. These experiments were repeated a minimum of three times, with at least three different preparations of Nb-G5.

#### Statistical Analysis of Biolayer Interferometry Fits

Data were reference-subtracted and aligned with each other in the Octet Data Analysis software (FortéBIO). Sensograms were fit with a 1:1 binding model to obtain kinetic binding constants. Equilibrium dissociation constant (*K*_D_) values were calculated from the ratio of *K*_off_ to *K*_on_. Global fits with an *R* ^2^ higher than 0.98 were considered acceptable.

### Crosslinking Reactions

Crosslinking reactions were conducted in the following manner. azidoPhe-incorporated protein and target were combined in a 1:6 molar ratio in 50 mM Tris, pH 7.5, 150 mM NaCl for a total reaction volume of 50 μL. This solution was allowed to incubate for 30 min on ice, and then exposed to UV light (254 nM) *via* a UVP Inc. Model UVGL-25 Multiband UV-254/366 nm Mineralight Lamp for 5 minutes in a 96 well UV-transparent plate also on ice. After exposure to UV light, the solutions were removed from the wells and mixed with SDS sample loading buffer and analyzed with SDS-PAGE.

### Western Blots

Western blot samples were separated on 4–22% gradient SDS-PAGE gels, transferred to PVDF membrane (30 V, overnight) blocked with Licor blocking buffer in TBST, and probed with anti-His (Takara, 1:1,000), or anti-V5 (Invitrogen, 1:500) primary antibodies rocking for 16 h at 4°C. After rinsing three times with TBST, the membranes were than incubated with Li-Cor IRDye 800CW Goat anti-rabbit or anti-mouse IgG (1:10,000) secondary antibody, rocking for 1 h at room temperature, and washed three times for 5 min in TBST. The membrane was then scanned using a Li-Cor Odyssey 9,120 Imaging System. If antibody overlays were desired, after imaging, the additional antibody was added to the membrane and incubated for 1 h at room temperature. After incubation, an appropriate secondary antibody was added, and the blot visualized as described above.

## Results

### Strategy and nitroTyr Protein Generation

#### Strategy

With the goal of generating a Nb that is either selective for a nitration site in a specific protein or one that binds nitroTyr regardless of protein context, we chose to immunize an animal with peptides that represented multiple nitrated targets (general and protein-specific) and use the results of a preliminary serum screen to decide the best target(s) to pursue. Per usual protocols (Vincke et al., 2012), from the blood of a seropositive immunized animal, we generated a phage-display library from peripheral blood lymphocyte RNA and carried out phage display selections with select nitrated target proteins. The selected nitroTyr-specific Nbs were then characterized for their affinity and selectivity using both semi-quantitative (ELISA, dot-blot) and quantitative (biolayer interferometry) methods.

#### NitroTyr-Protein Targets for Selections and for Immunizations

**FIGURE 1:**
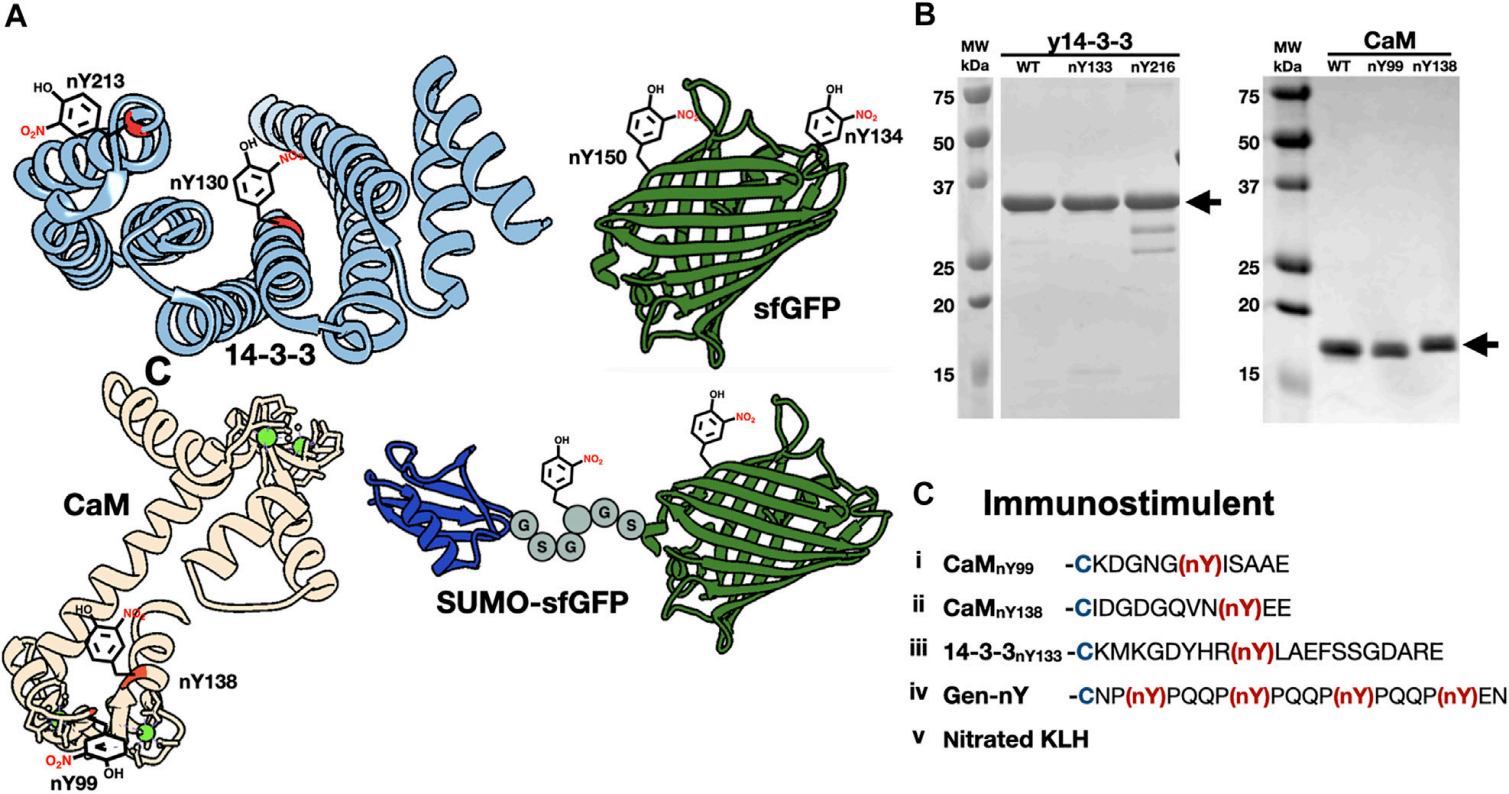
The generation of nitrated molecules for Nb selections and evaluations. **(A)** Structures of nitrated targets used in this study and generated with Chimera. Nitration sites are indicated with nitroTyr drawing. (PDB ID-2BQ0, 3CLN, 2B3P). **(B)** SDS-PAGE analysis of purified nitrated targets. **(C)** Schematic of five immunogens included in injection mixture. Peptides i-iv are conjugated to KLH with a N-terminal cysteine residue (blue). NitroTyrs in peptides are indicated in red.

We selected signaling hub proteins 14-3-3 and CaM as nitroTyr-protein targets (Figure 1A). The yeast ortholog of 14-3-3 (commonly known as BMH1 and referred to here as “y14-3-3” for clarity) was used initially as it is a functional model for human 14-3-3 [(Clapp et al., 2012), ∼55% identity, h14-3-3] and we had established expression and purification systems for it. While proteomics studies have identified multiple sites of *in vivo* nitration of y14-3-3 (Nuriel et al., 2015; Pennington et al., 2018), we initially focused on Y133, which is a key part of pSer/pThr binding site and fully conserved in all eukaryotic 14-3-3 proteins. For CaM, we focused on Y99 which when nitrated serves as a biomarker of oxidative stress (Smallwood et al., 2003) and Y138 which both enhances CaM binding to eNOS and has been shown to be transient in activated macrophages (Porter et al., 2020). These two sites also provide contrasting protein contexts, as Y99 is found in the less solvent-exposed globular Ca^2+^-binding domain and Y138 is contained in a conformationally dynamic region (Smallwood et al., 2007; Piazza et al., 2012; Porter et al., 2020) (Figure 1A). For our immunization series, we designed peptides encompassing all three of these nitration sites along with an additional peptide containing several nitroTyrs to enhance the chance of obtaining Nbs that were generally specific for nitroTyr. With these peptides, we also included in the immunization series chemically nitrated keyhole limpet hemocyanin (KLH) (Figure 1C) which emulated the successful strategy employed for the original nitroTyr-Ab generation (Beckmann et al., 1994).

#### NitroTyr-Proteins for Further Binding Characterization

In addition to nitrated immunogens and the specific target proteins to be used in selections, we generated some nitroTyr-proteins to further characterize the specificities of the generated Nbs (Figure 1A and Table 1). To that end, we identified y14-3-3 Y216 whose nitration is also physiologically relevant and is more solvent-exposed than Y133. Additionally, the human variants of 14-3-3β (nY130 and nY213) were used because their nitration is more relevant to human disease. We further made superfolder green fluorescent proteins (sfGFPs) with nitroTyr inserted in the place of N150 or D134 to study the ability of the Nb to bind a nitroTyr that juts out from a smooth protein surface (site 150) as compared to a nitroTyr located in the loops of the barrel (site 134). And finally, to evaluate the effects of having multiple sites of nitration contained in a single protein, we created a SUMO-linker-sfGFP containing two sites of nitration. Using GCE, we produced all full-length, homogenously nitrated proteins, purified to >95% purity with yields ranging from 30–45 mg/L of culture (Figure 1B). The fidelity of site-specific nitroTyr incorporation was verified by mass spectrometry, with correct masses obtained for both unmodified and nitrated targets (Supplementary Figure S1). In total, nine specific nitroTyr proteins were produced for generating and testing Nbs (Table 1).

**TABLE 1:** NitroTyr proteins produced for Nb evaluation.

Protein	Nitration sites	Biological significance	Structural context	Ref
y14-3-3	Y133	Nitrated *in vivo*	Key component of pSer/pThr recognition triad	Zhao et al. (2017), Pennington et al. (2018)
y14-3-3	Y216	Nitrated *in vivo*	Solvent exposed	Nuriel et al. (2015), Pennington et al. (2018)
h14-3-3	Y130	Nitrated *in vivo*	Key component of pSer/pThr recognition triad	Zhao et al. (2017), Pennington et al. (2018)
h14-3-3	Y213	Nitrated *in vivo*	Solvent exposed	Nuriel et al. (2015), Pennington et al. (2018)
CaM	Y99	Nitration site is a target for denitrase	Found in globular Ca^2+^ binding domain	Corti et al. (1999), Smallwood et al. (2007)
CaM	Y138	Nitration at site modulates signaling behavior	Found in a conformationally dynamic region	Mukherjea et al. (1996), Porter et al. (2020)
GFP	Y134	None	In protein loop	N/A
GFP	N150	None	On side of GFP barrel	N/A
SUMO-GFP	Link, N150 (2x nitroTyr)	None	Dual nitration sites; one on a disordered linker	N/A

### Identification of Two Promising Nbs Selected to Recognize 14-3-3_nY133_

#### Immune-Serum Generation and Screening

**FIGURE 2:**
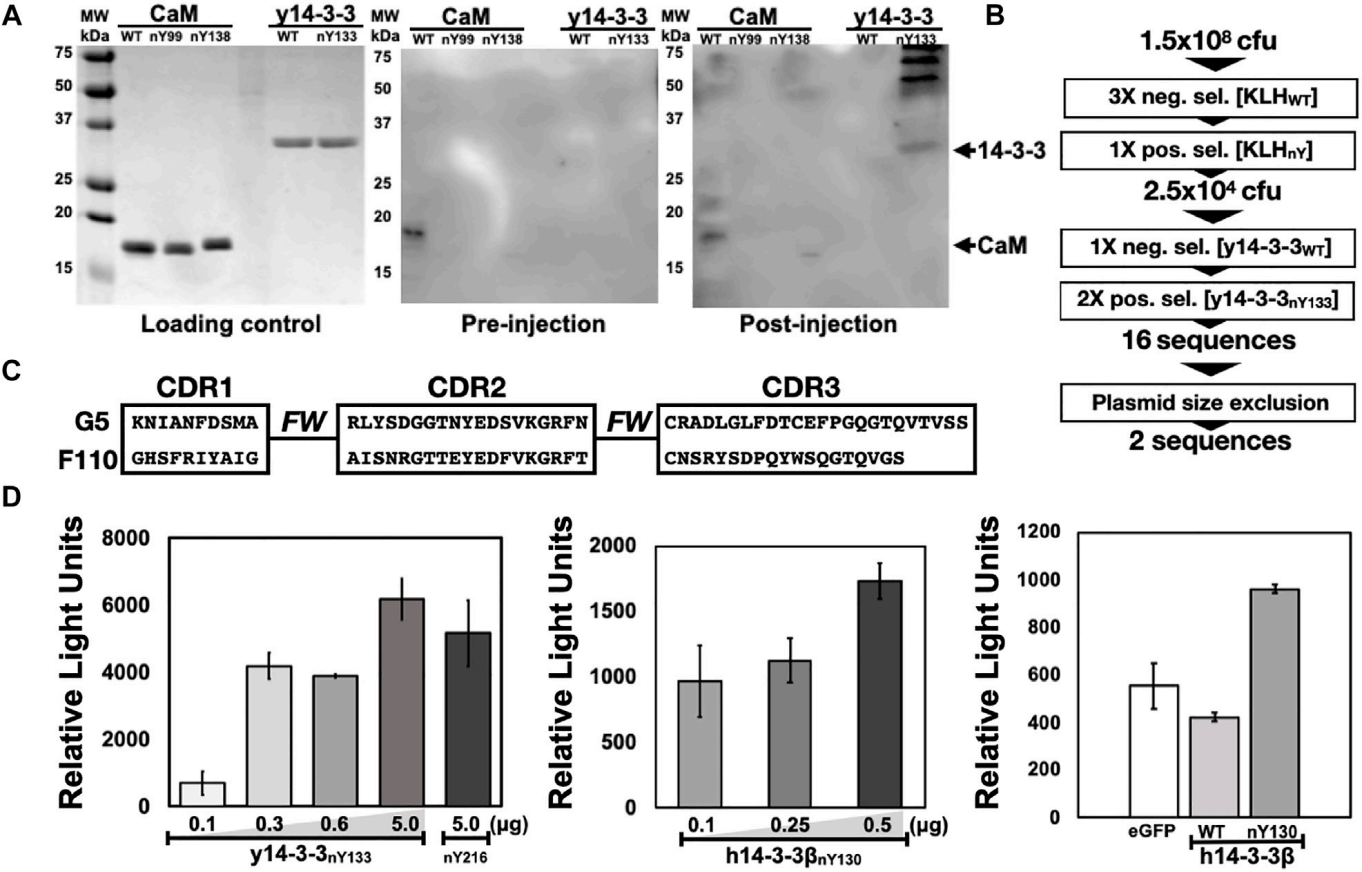
Selection and preliminary validation of Nb-G5. **(A)** Serum analysis of injected animal against nitrated targets. Five protein targets (WT and nitroTyr, listed at top of gel) were resolved with SDS-PAGE. Gels were either stained (Loading control) or transferred to a PVDF membrane and incubated with serum from before injections (pre-injection) or after (post-injection). These membranes were probed with HRP-conjugated anti-llama primary and visualized. Arrows indicate the expected migration of protein targets. **(B)** Schematic of selections with full-length, folded nitroTyr 14-3-3. Included is the number of cfu or sequences obtained after significant rounds. **(C)** CDR regions of Nb-G5 and Nb-F110. “FW” indicates the framework region of the Nbs. Full sequences are found in Supplemental Figure S2. **(D)** ELISA of Nb-G5 nitrated 14-3-3. Increasing amounts of His-tagged y14-3-3β nitroY133 (left panel) 14-3-3β nitroY130 (middle panel) were immobilized on a nickel-coated ELISA plate and incubated with NB-G5 (25 μg/ml) in TBST followed by incubation with HRP-conjugated anti-llama primary antibody. Right panel: His-tagged sfGFP, 14-3-3β WT and 14-3-3β nitroY130 (0.25 μg). were immobilized on a nickel-coated ELISA plate and incubated with NB-G5 (25 μg/ml) in TBST followed by incubation with HRP-conjugated anti-llama primary antibody.

All five nitrated immunogens were simultaneously injected into an alpaca following a standard immunization procedure (*Materials and Methods*). To monitor the production of Camelid antibodies that target nitroTyr-proteins, pre- and post-injection sera (collected before and after full immunization) were incubated with membrane-immobilized y14-3-3 and CaM targets. Development of the membranes revealed bands in the lane containing y14-3-3_nY133_ and a faint band in a lane containing CaM_nY138_, none of which appear on the pre-serum membrane providing evidence that antibodies specific to nitrated targets had been produced (Figure 2A). Additional prominent higher molecular weight bands were also visible in the y14-3-3_nY133_ lane; these may represent camelid antibody affinity for dimerized forms of 14-3-3. Due to the intensity of the y14-3-3_nY133_ bands compared to the other targets (CaM_nY99_ and CaM_nY138_), we sought to develop a protein and nitration site-specific Nb and pursued selections against y14-3-3_nY133_.

#### Library Generation and Phage Display Selections Using y14-3-3_nY133_

To generate a phage display library, the total RNA from peripheral blood lymphocytes (PBLs) after the immunization procedure were used to make Nb-encoding cDNA. The cDNAs were amplified and subcloned into the phagemid vector pSEX81, generating a phage display library. With this library, we performed seven rounds of phage display selections (Figure 2B). The first three rounds were negative selections using KLH as the target, designed to exclude anti-KLH library members. Then, mimicking the process that yielded the initial nitroTyr-Abs (Beckmann et al., 1994), we carried out a positive selection with nitroTyr-KLH to ensure that remaining Nbs had some direct recognition of nitroTyr. These four rounds of selection diminished the library size from 1.5 × 10^8^ cfu (colony forming units) to 2.5 × 10^4^ cfu (Figure 2B).

We then panned for library members that were selective for y14-3-3_nY133_ by using one round of negative selection with wild type y14-3-3, followed by two rounds of positive selections with y14-3-3_nY133_ (Figure 2B). After a plasmid size-exclusion step to eliminate truncated Nbs, two full-length Nbs were obtained: Nb-G5 and Nb-F110 (Figure 2C, Supplementary Figure S2). Expression and purification of Nb-G5 yielded protein sufficient for downstream characterization (Supplementary Figure S3) but we were unable to identify suitable *E. coli* expression strategies for Nb-F110. As a result, we moved forward with evaluating only Nb-G5.

### Characterization of the Binding Properties of Nb-G5

#### Semi-Quantitative Survey of Nb-G5 Binding to 14-3-3 Protein Forms

Binding of Nb-G5 was qualitatively evaluated with both dot-blot assays and enzyme-linked immunosorbent assays (ELISA). With the dot-blot assay, we evaluated Nb-G5 selectivity and Nb binding sensitivity. Nitrated targets y14-3-3_nY133_ and y14-3-3_nY216_ along with y14-3-3ß_WT_ were dotted in varying amounts onto a membrane and probed with Nb-G5; binding was observed for both nitroTyr proteins but not y14-3-3ß_WT,_ indicating a selectivity for nitrated protein (Supplementary Figure S4). y14-3-3_nY216_ was included to see if Nb-G5 possessed any sensitivity for nitration independent of the protein context of site nY133. Having at this point successfully expressed the more biomedically relevant nitrated human 14-3-3ß (*h*14-3-3ß) isoforms, we also probed these and found they were recognized with a similar level of sensitivity (Supplementary Figure S4).

The range of selectivity and sensitivity of Nb-G5 to immobilized, folded protein in solution was assayed by ELISA. For both the yeast and human 14-3-3 variants, significant signal above background was observed, down to 0.1 µg of immobilized nitrated 14-3-3. The selectivity of Nb-G5 for nitroTyr was evaluated by assaying immobilized 14-3-3ß_WT_ and sfGFP (as a negative control) alongside h14-3-3ß_nY130_. Significant signal for h14-3-3ß_nY130_ over sfGFP and h14-3-3ß _WT,_ which both exhibited a similar level of background was also observed by ELISA (Figure 2D). Based on this, we decided to carry out quantitative binding studies with the h14-3-3ß forms rather than with the y14-3-3 forms.

#### Quantitative Analyses Nb-G5 Affinity and Selectivity

**FIGURE 3:**
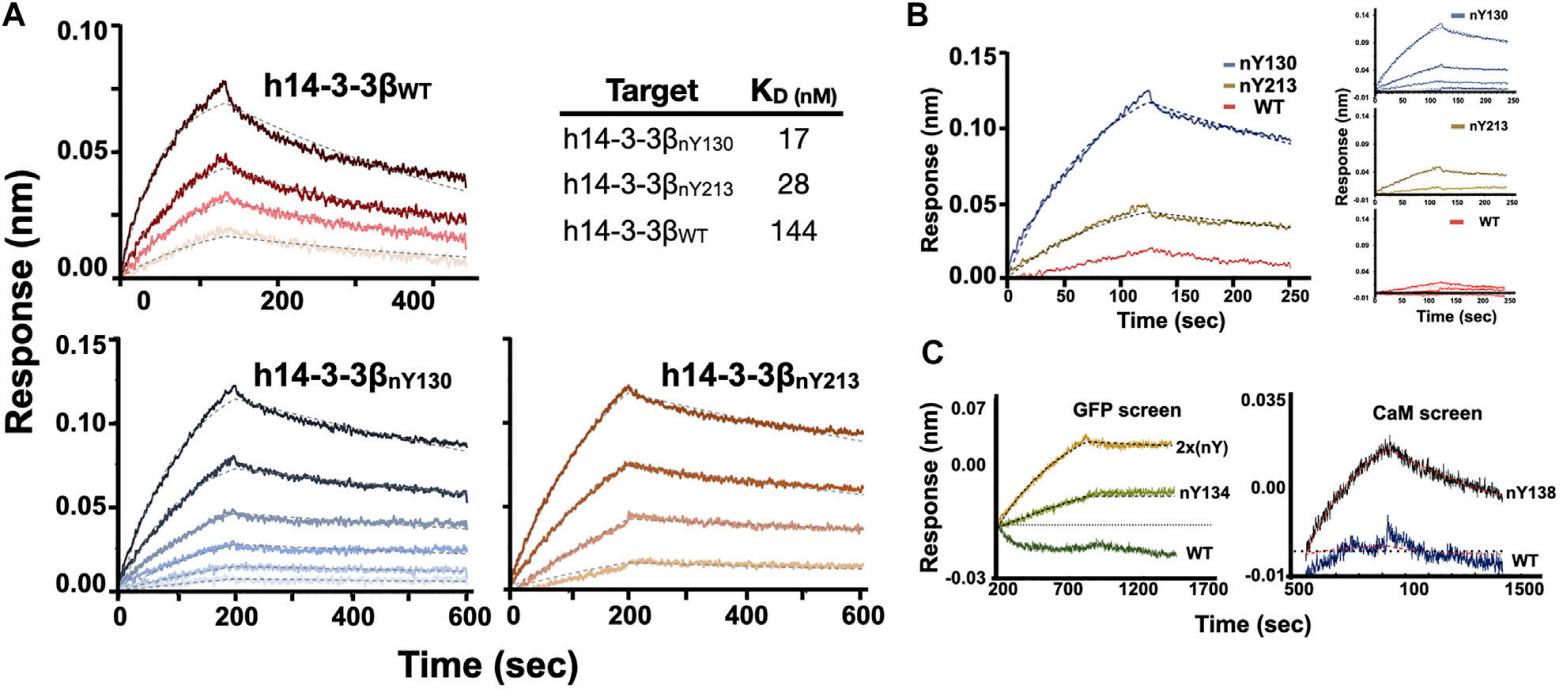
BLI analysis of Nb-G5 binding ability. **(A)** BLI sensograms of Nb-G5 against nitrated and WT 14-3-3β in 0.2% BSA. For global fits, concentrations of Nb-G5 against nitrated ranged from 25 to 200 nM for 14-3-3β_nY130_, 40–200 nM for 14-3-3β_nY213_ and 270–900 nM for 14-3-3β_WT_. K_D_ values for global fits are listed in the upper right. **(B)** BLI sensograms of Nb-G5 against nitrated and WT 14-3-3β in 1% BSA. Left panel: the sensograms for all three targets at 450 nM. Right panel: Global fits for Nb-G5 in 1% BSA ranging from 80 to 450 nM. **(C)** Left panel: Local fits of Nb-G5 (200 nM) against sfGFP_WT_, sfGFP_nY134_, and sfGFP_2xnY_. Right panel: Local fits of Nb-G5 (300 nM) against CaM_WT_ and CaM_nY138_.

Encouraged by our preliminary evaluation of Nb binding, we employed biolayer interferometry (BLI) to quantitatively assess the sensitivity and selectivity of Nb-G5. Avi-tagged h14-3-3ß variants (WT, nY130 and nY213) were immobilized onto BLI tips and incubated with Nb-G5 to measure the association and dissociation kinetics of binding. Global fits of the BLI sensograms in 0.25% bovine serum albumin (BSA) gave a K_D_ value for Nb-G5 against h14-3-3ß_nY130_ of ∼14 nM, a value that is an order of magnitude tighter than that obtained for Nb-G5 against h14-3-3ß_WT_ under the same assay conditions, and 2–5 times tighter than the K_D_ for h14-3-3ß_nY213_ (Figure 3A and Table 2). The main contributor to the observed differences in K_D_ is the dissociation rate, consistent with the nitroTyr protein forms making specific interactions with Nb-G5 that slow its dissociation (Table 2). To minimize the contribution of non-specific binding, the assays were also performed in 1% BSA. In these experiments, binding to h14-3-3ß_WT_ was almost completely ablated and kinetic constants could not be determined, although binding to h14-3-3ß_nY130_ was still observed (K_D_ ∼40 nM, Figure 3B). This is consistent with binding to WT protein being non-specific, whereas binding to both nitrated protein forms involving some level of specific recognition.

**TABLE 2:** Kinetic constants for Nb-G5:14-3-3 binding.

TARGET (in 0.2% BSA)	K_D_ (nM)	k_on_ (10^4^ M^−1^ s^−1^)	k_off_ (10^3^ s^−1^)	BSA
14-3-3 ***β***_nY130_	17 ± 3	2.2 ± 0.2	0.33 ± 0.02	0.2%
14-3-3 ***β***_nY213_	28 ± 2	2.6 ± 0.1	0.73 ± 0.02	0.2%
14-3-3 ***β***_WT_	144 ± 8	2.1 ± 0.6	2.3 ± 0.5	0.2%

TARGET (1.0% BSA)	K_D_ (nM)	k_on_ (10^4^ M^−1^ s^−1^)	k_off_ (10^3^ s^−1^)	BSA
14-3-3 ***β***_nY130_	36 ± 3	3.2 ± 0.1	1.19 ± 0.07	1.0%
14-3-3 ***β***_nY213_	110 ± 3	2.05 ± 0.02	2.26 ± 0.05	1.0%
14-3-3 ***β***_WT_	Undeterminable	Undeterminable	Undeterminable	1.0%

To further assess the relative contributions of the nitroTyr group itself *versus* the protein context to the binding of Nb-G5 for nitrated targets, we tested Nb-G5 binding with proteins containing nitroTyr in different protein locations (Figure 1A). Proteins included three CaM forms (WT, nY99, nY138 in 0.05% Tween) and four sfGFP forms [WT, nY134, nY150, SUMO-link-sfGFP (2x nitroTyr) in 0.2% BSA, Table 1]. Although none of these proteins bound as well as either of the nitrated forms of h14-3-3ß, local fits revealed some binding to both CaM_nY138_ (Figure 3C panel A), sfGFP_nY134_ and SUMO-link-sfGFP (2x nitroTyr) (Figure 3C panel B) for these targets at high Nb-G5 concentrations (>200 nM). These interactions generated K_D_ values >100 nM, and no notable binding to the wild type proteins or to the nitrated targets GFP_nY150_ or CaM_nY99_ was observed (Supplementary Figure S5).

### Nb-G5 Nanobody as Basis for Creating nitroTyr Specific Cross-Linking Tools

#### Identification of Potential Sites and Construct Design for Crosslinking Experiments

**FIGURE 4:**
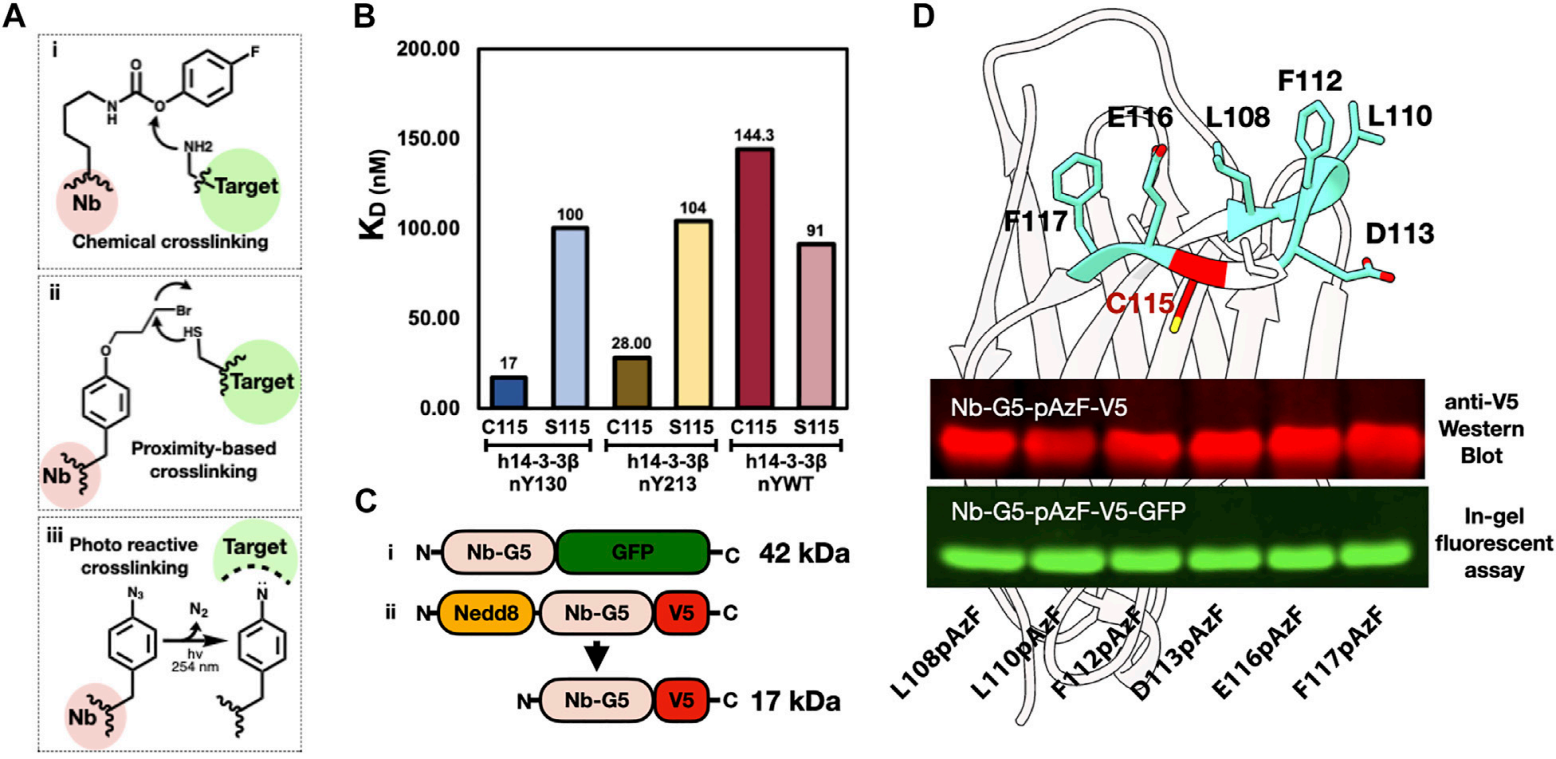
Generating an anti-nitroTyr cross-linking tool. **(A)**. Examples of three common crosslinking strategies: (i) Chemical crosslinkers, such as the carbamate-containing ncAA FPheK, display a high reactivity to proximal nucleophilic residues (e.g., Lys, Cys, and Tyr) under weakly basic conditions (Xuan et al., 2017); (ii) Proximity-based crosslinkers, such as the haloalkane ncAA shown, react with Cys residues in the target protein facilitated by an increased local effective concentration (Xiang et al., 2014); and (iii) Photoreactive crosslinkers, such as benzophenones, diazirines, and azides (as in the azidoPhe ncAA shown), contain UV-induced cross-linking moieties that react with diverse proximal groups (Hoffmann 2020). **(B).** K_D_s of Nb-G5 and Nb-G5 (C115S) against nitrated and WT 14-3-3 are shown. **(C)**. Constructs of Nb-G5-GFP (i) and Nedd8-Nb-G5-V5 (ii). **(D)**. A model of Nb-G5, generated by ABodyBuilder: automated antibody structure predictor (Leem et al., 2016). The CDR3 loop unpaired cysteine (C115) is indicated in red and the adjacent resides individually substituted with azidoPhe are colored teal and labeled. An anti-V5 western blot image (top panel) and an in-gel fluorescence assay (bottom panel) show both kinds of azidoPhe-encoded Nb-G5 constructs expressed well.

As noted in the introduction, generating covalent Nb binders (i.e., “GlueBodies”) through incorporating ncAAs with crosslinking abilities (ncAA-CL), is a means to target proteins for degradation (Zhang et al., 2021). Nbs that target oxPTMs for degradation would be powerful tools for discovering possible physiological changes triggered by specific oxidized proteins. A variety of ncAA-CL can be incorporated by GCE, including chemically reactive, proximity-induced and photo-reactive ncAA-CLs (Figure 4A). Because the GCE machinery for photocrosslinking ncAA-CL 4-azido phenylalanine (azidoPhe, pAzF) is robust, the ncAA is commercially available and the crosslinking assays are simple to perform, we decided to investigate the suitability of Nb-G5 as a covalent crosslinker by the installation of azidoPhe into Nb-G5 with GCE.

We first sought to identify locations on Nb-G5 near the edge of the paratope, so that upon binding they would be close to the target, yet not interfere with Nb binding itself. We noticed the CDR3 loop of Nb-G5 contained an unpaired cysteine residue which is unusual in Nbs (Bannas et al., 2017) and so we speculated this Cys was part of the antigen binding site. To evaluate its contribution, we generated a C115S mutant of Nb-G5 and observed by BLI that the binding to h14-3-3ß_nY130_ decreased by six-fold, and that the selectivity for h14-3-3ß_nY130_ over h14-3-3_WT_ was lost (Figure 4B).

We created two different strategies to visualize the crosslinked material (Figure 4C). The first was appending a C-terminal GFP fusion to the Nb-G5, which would give a sensitive fluorescent signature to the Nb and allow CL-dependent mass shifts to be easily observed with in-gel fluorescence. Concerned that a bulky GFP tag could impact the binding of Nb-G5, we also designed a construct with a smaller C-terminal V5 tag, which could be visualized by Western blotting (Figure 4C, “ii”). Also, since we expected the Nb-G5 incorporating azidoPhe would express less well, we sought to enhance the expression through adding a cleavable N-terminal bdNEDD8 solubility tag (Pleiner et al., 2018). With the systems designed, we selected six potential sites flanking C115 for azidoPhe incorporation, and all six sites for both designs (GFP and V5) yielded sufficient cleaved, azidoPhe-containing Nb-G5 for crosslinking experiments (Figure 4D, Supplementary Figure S6).

#### Crosslinking Attempts With h1413-3ß_nY133_ and CaM_nY138_

As nitroTyr-protein targets, we selected the two that were bound best by Nb-G5: 14-3-3_nY133_ and CaM_nY138_. Both targets possess C-terminal His tags, allowing them to be detected with Western Blot. Screening of the six azidoPhe sites with both nitrated targets for their ability to yield nitroTyr-dependent crosslinked products yielded disappointing results (Supplementary Figures S7, S8). Some bands appear at the correct molecular weight range for pAzF113 with the CaM target and pAzF108 and pAzF110 for the 14-3-3 target, but even for these the amount of CL product is minimal and masked by the presence of non-specific crosslinked products. In addition, the photoactivation appears to dimerize the nitroTyr-containing target protein, further complicating analysis and making the approach with this Nb-G5 and azidoPhe photocrosslinker challenging to implement in a more complex environment (live cell).

## Discussion

With the aid of GCE technology, we show it is possible to generate a Nb selective for an oxidatively-modified protein target, opening the doors to dynamic studies of oxPTM-impacted proteins in living cells. This technology is not limited to oxPTMs, as GCE can provide site-specifically modified protein for many biologically important PTMs, including phosphoserine (Rogerson et al., 2015; Zhu et al., 2019) and acetyl lysine (Neumann-Staubitz et al., 2021). This allows, for the first time, a rigorous characterization of the site and protein specificity of Nbs developed against these targets contributing to both the engineering of nanobodies for specific purposes as well our general knowledge of Nb-target recognition.

Starting from the serum of an animal injected with a mixture of five nitrated immunogens, we developed an anti-nitroTyr nanobody library and performed phage-display selections for the nitrated target 14-3-3_nY130_, obtaining the sequence for a single soluble nanobody, Nb-G5. BLI analysis of Nb-G5 with 14-3-3_nY130_ revealed a 10-fold selectivity for 14-3-3_nY130_ over 14-3-3_WT_ in 0.2% BSA solutions, exhibiting a modest K_D_ of ∼14 nM. In 1% BSA solutions, Nb-G5 exhibited a higher K_D_ but complete selectivity for 14-3-3_nY130_ over 14-3-3_WT_. Nb-G5 also showed some weak yet selective binding to other non-14-3-3 nitrated targets, which may indicate that the Nb is interacting with the nitroTyr molecule itself. This weak binding was site-dependent and appeared to be strongest when the nitroTyr molecule was exposed, but still surrounded by ample protein context.

After obtaining some information about a cysteine that is critical to Nb selectivity, we were able to site-specifically install the ncAA azidoPhe in the CDR3 region of Nb-G5. Using robust GCE machinery for azidoPhe, all azidoPhe sites were successfully installed at reasonable yields allowing us to easily screen the library against two nitrated targets for crosslinking. However, the amount of crosslinked product produced was small, and the presence of nitroTyr in proteins appeared to increase the propensity of the nitrated proteins to crosslink under UV exposure, limiting the suitability of this specific crosslinking strategy for nitroTyr targets. Nitrotyrosine increases the UV absorbance of the target proteins (Crow and Beckman 1995) and appears to be competing with the desired photocrosslinking pathway. With GCE, we are not limited to one type of crosslinking chemistry and could utilize crosslinkers that instead are proximity-based and operate independently of UV irradiation, including ncAA with bromoalkyl moieties (Cigler et al., 2017). These would be well-suited for Nbs targeting chemically labile modifications which may be more sensitive to UV irradiation such as those installed under conditions of oxidative stress. Regardless, we were encouraged by the ability to form ncAA-CL encoded-Nbs, showing that GCE-technology is well suited to be implemented in every step of Nb development, from selections to additional functionalities.

The mid-nanomolar K_D_ obtained for NB-G5 with no additional rounds of affinity maturation is promising, however, Nbs that are designed to be employed as intracellular tools require low to sub-nanomolar affinities for their targets (Mahajan et al., 2018). Originally, when we were performing the selections, we included a positive selection step with nitrated KLH to emulate the strategy that produced the first anti-nitroTyr antibodies. It may be that more selective binders for 14-3-3nY were excluded from the set in that KLH_nY_ positive selection step, although Nb-G5 has slight general specificity. The pre-KLH positive step library still exists, and it represents a valuable resource in itself that could be used to select for Nbs against other nitroTyr proteins, or a tighter 14-3-3nY133 binder. With respect to the selections themselves, Nb-selection technology is rapidly improving. With GCE-produced proteins and multiple rounds off affinity maturation, the development of anti-oxPTM Nbs with sub-nanomolar affinities, appropriate of intracellular interrogation, are within reach. Such Nbs will be powerful tools that will open up a window into the complex intracellular dynamics of oxidatively modified proteins and their impacts.

## Data Availability

The original contributions presented in the study are included in the article/Supplementary Materials, further inquiries can be directed to the corresponding author.
