# Supplementary material for: Creating a Selective Nanobody Against 3-Nitrotyrosine Containing Proteins
Source: Front Chem. 2022 Feb 21;10:835229. doi: 10.3389/fchem.2022.835229 (PMC8899190; doi:10.3389/fchem.2022.835229)
Supplement: Supplementary file 1 [file DataSheet1.PDF]

Supplementary Material

Creating a selective nanobody against 3-nitrotyrosine containing proteins

Elise M. Van Fossen[1], Sonia Grutzius[1] Carl E. Ruby[2], Dan Mourich[2], Chris Cebra[2], Shay Bracha[3], P. Andrew Karplus[1], Richard B. Cooley[1], Ryan A. Mehl[1]

[1]Oregon State University, Department of Biochemistry and Biophysics, 2011 Agricultural and Life Sciences, Corvallis, OR 97331

[2]Oregon State University, Carlson College of Veterinary Medicine, Magruder Hall, 700 SW 30th St, Corvallis, OR 97331

[3]Texas A&M College of Veterinary Medicine & Biomedical Sciences, Department of Small Animal Clinical Sciences (VSCS), 4474 TAMU, College Station, TX 77843

*** Correspondence:**
Ryan Mehl
Ryan.Mehl@oregonstate.edu

Table of Contents:

Supplementary Figures 1-8

Supplementary Tables 1-3

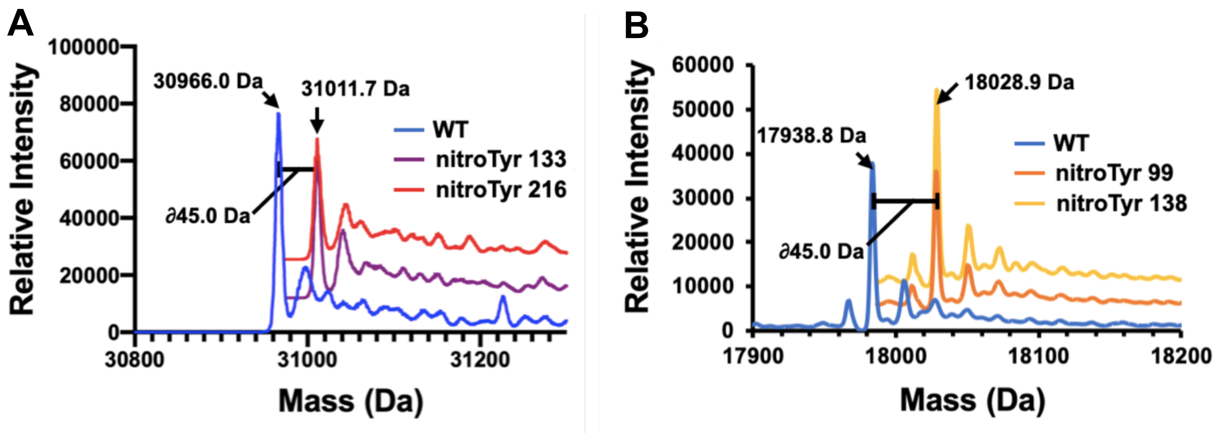

Supplementary Figure 1. Whole protein mass spectra show homogeneous incorporation of nitroTyr into 14-3-3 and CaM. A. Mass spectra of y14-3-3_{WT}, y14-3-3_{nY133} and y14-3-3_{nY216}. B. Mass spectra of CaM_{WT}, CaM_{nY99} and CaM_{nY138}. Indicated are the measured masses.

Nb-G5
MAQVQLQESGGGLVQPGGSLRLSCAASKNIANFDSMAWYRRSPGKERDLVARLYSDGGTN
YEDSVKGRFNISTDIEKNTVYLQMNNLKLEDTARYYCRADLGLFDTCEFPGQGTQVTVSS

Nb-F110
MAQVQLQESGGGLVQAGGSLTLSCTPSGHSFRIYAIGWYRQVPGNQRELVAAISNRGTTEY
EDFVKGRFTISRDNAKKTAYLQMNNLRPDDTAVYYCNSRYSDPQYWSQGTQVGS

Supplementary Figure 2. Sequences of Nb-G5 and Nb-F110

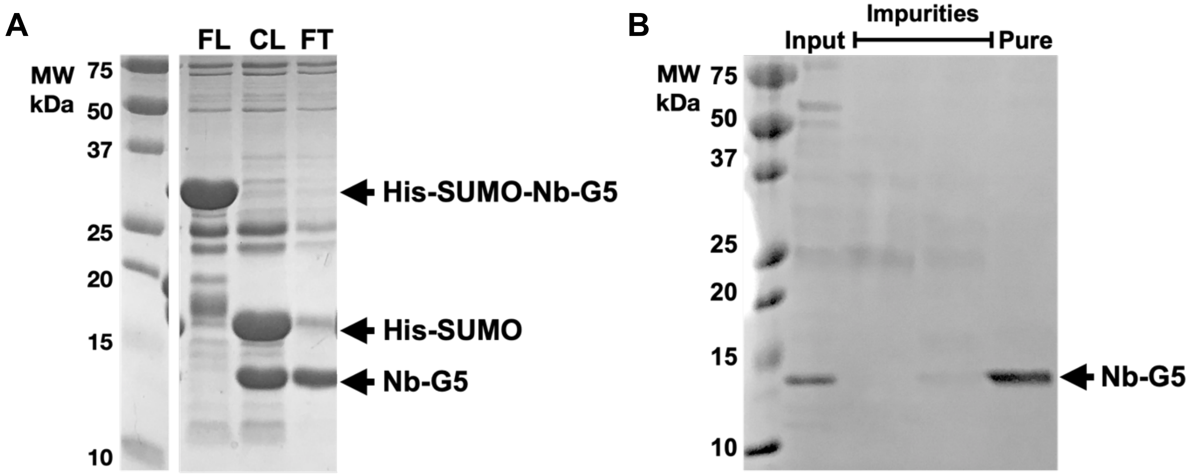

Supplementary Figure 3. SDS-PAGE analysis of Nb-G5 expression. A. Purification process of Nb-G5 shown by SDS-PAGE gel electrophoresis. FL - Full-length construct before cleavage, CL - cleavage products after the addition of ULP, FT - Cleaved Nb-G5. B. SEC purification of Nb-G5 with final pure Nb-G5

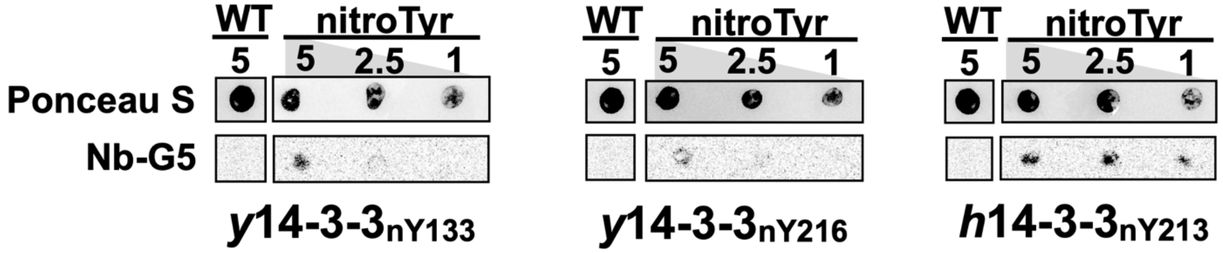

Supplementary Figure 4. Dot-blot of nitrated proteins incubated with Nb-G5 and probed with HRP-conjugated anti-llama primary antibody. Proteins (5.0 ug - 1.0 ug) were blotted onto Immobilon PVDF and incubated with Nb-G5 (1:3,000) overnight at 25 ˚C, followed by an overnight incubation with HRP-conjugated anti-llama primary antibody (1:1,000). Blot was visualized with Western Lightning Plus ECL.

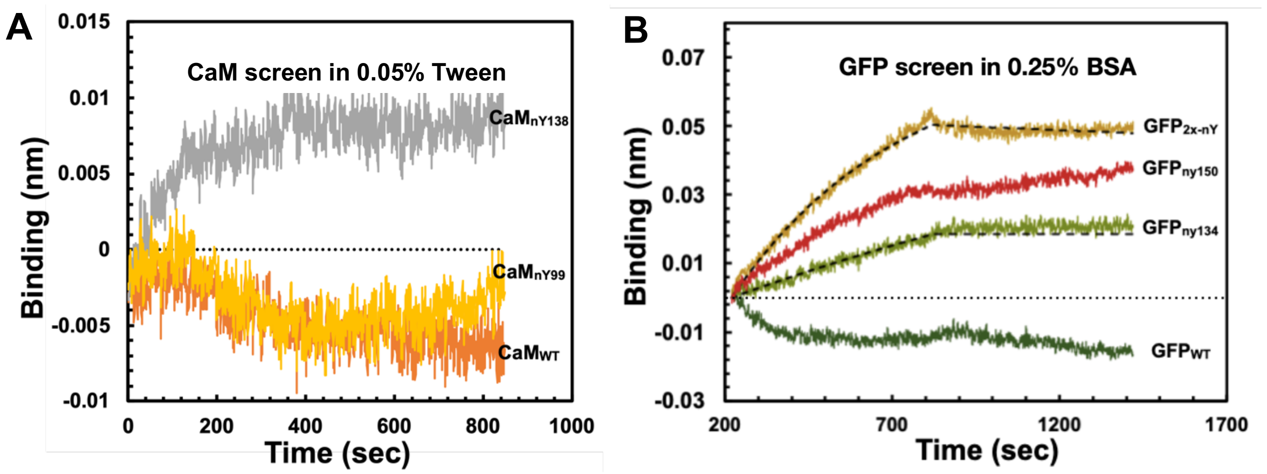

Supplementary Figure 5. BLI sensograms of Nb-G5 targeted to nitrated and WT non-14-3-3 targets. A. CaM selectivity screen with Nb-G5 (100 nM) in TBS with 0.05% Tween. B. GFP screen with Nb-G5 (200 nM) in TBS with 0.25% BSA.

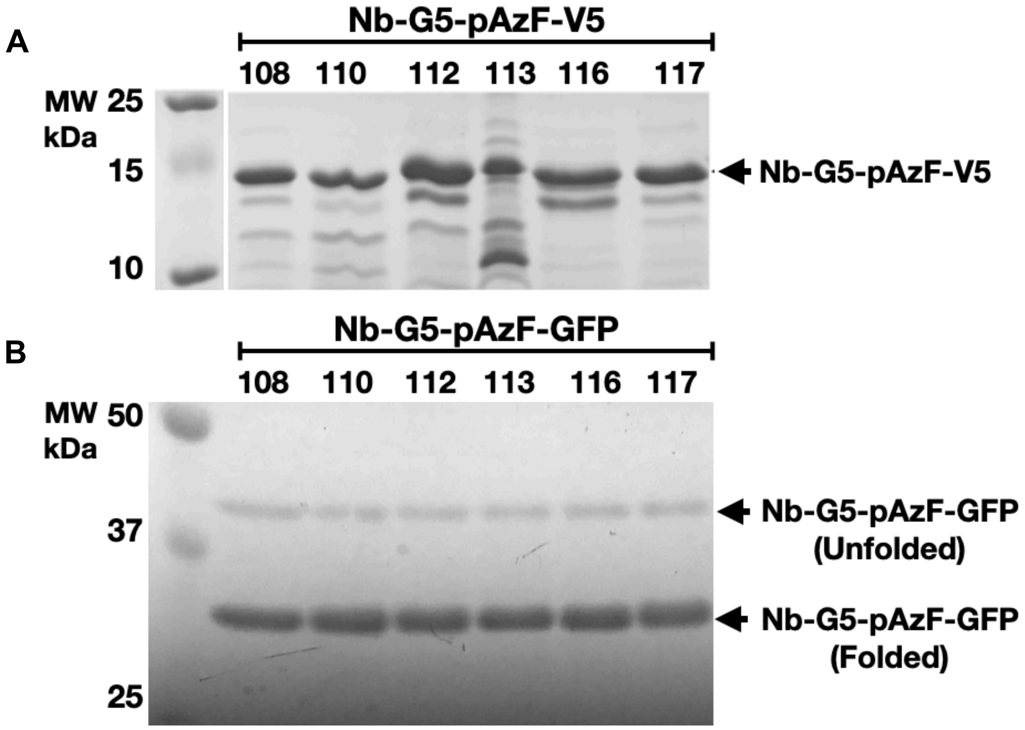

Supplementary Figure 6. Generation of crosslinking reagents. A. pAzF-incorporated Nb-G5 constructs analyzed with SDS-PAGE. The sites of incorporation are listed at the top of the gel. B. Native SDS-PAGE analysis of all Nb-G5-pAzF-GFP constructs. Some unfolded protein migrates at the expected MW of ~45 kDa, while the folded constructs move quicker through the gel, with an apparent MW of ~32 kDa.

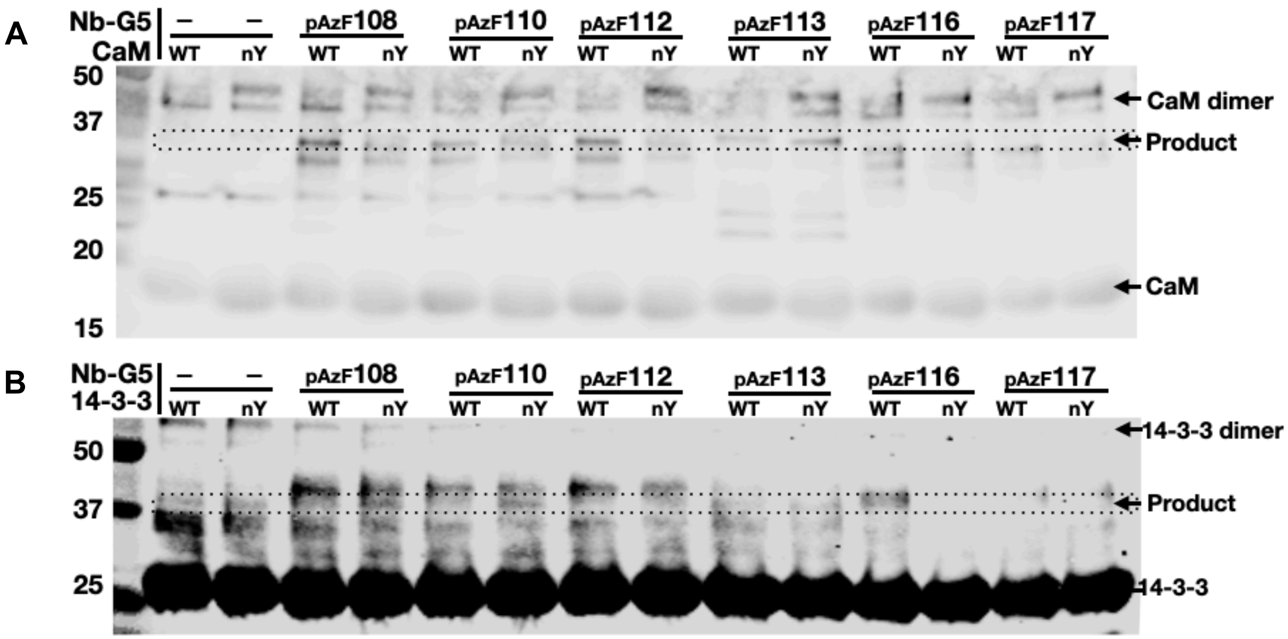

Supplementary Figure 7. Evaluation of crosslinking reagents. A. Anti-His blot of the results of library screen with CaM_{nY138}. Dotted boxes indicate the expected MW of product. Different species in the reactions are indicated by arrows. B. Anti-His blot of the results of library screen with h14-3-3_{nY130}. Dotted boxes indicate the expected MW of product. Different species in the reactions are indicated by arrows, including dimerized 14-3-3 and CaM.

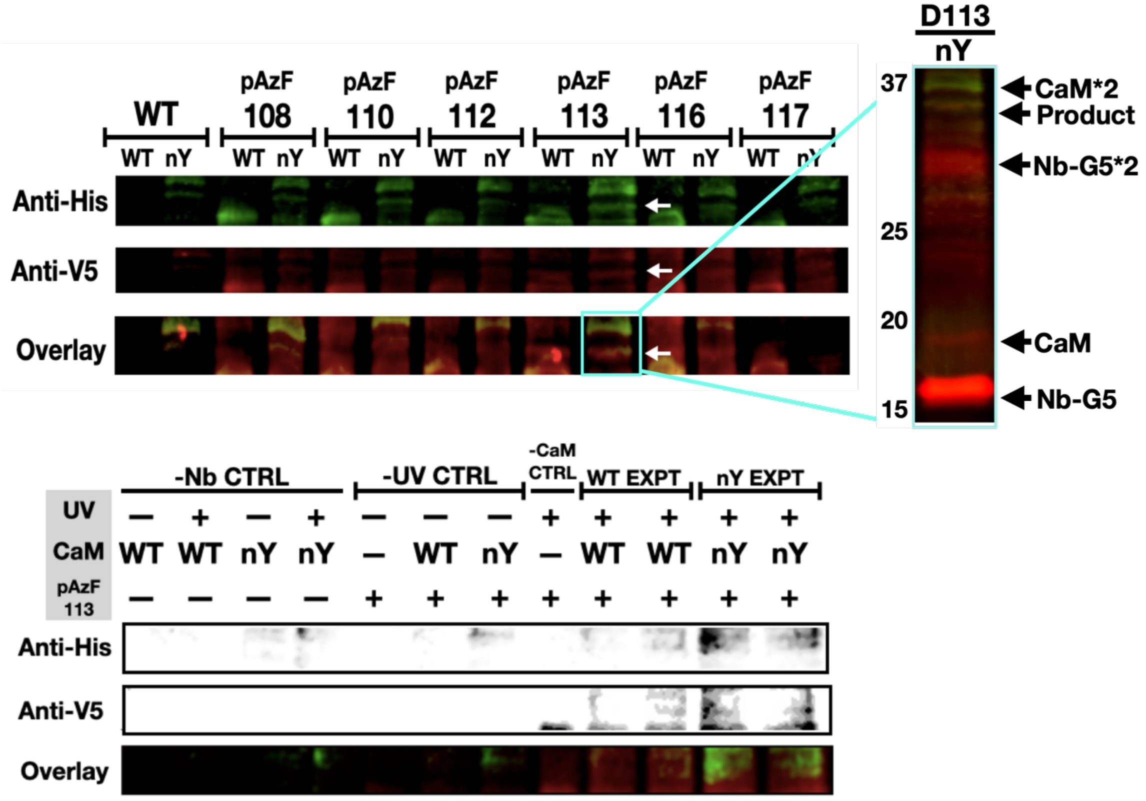

Supplementary Figure 8. Evaluation of site Nb-G5-pAzF113-V5 with additional controls. A. Cross-linking screen of Nb-G5 with CaM. White arrows indicate potential product formation. Entire gel slice of the blue box is shown to the right with black arrows indicating the reaction participants, including dimerized CaM. B. Further evaluation of site pAzF-113 with additional controls. Product should only appear in lanes with +UV, +nY and + pAzF-113

No.	Plasmid Name	Promoter(s)	Ori	Res.	Primers	Template	Backbone	Ref
1	pDULE2-nYRS-A7	lpp(aaRS)<br>lpp(tRNA)	p15	Spec	---	---	---	(Beyer, Hosseinzadeh et al. 2020)
2	pDULE2-pCNF	lpp(aaRS)<br>lpp(tRNA)	p15	Spec	---	---	---	(Miyake-Stoner, Miller et al. 2009)
3	pBAD-His-SUMO-Nb-G5	araBAD	pBR322	AMP	1,2	---	pBAD (NcoI/XhoI)	This Study
4	pBAD-His-SUMO-Nb-F110	araBAD	pBR322	AMP	1,2	---	pBAD (NcoI/XhoI)	This Study
5	pBAD-His-NEDD8-Nb-G5-V5	araBAD	pBR322	AMP	3-6	2	pBAD (NcoI/XhoI)	This Study
6	pBAD-His-NEDD8-Nb-G5-V5 [L108TAG]-V5	araBAD	pBR322	AMP	3,6,7,8	4	pBAD (NcoI/XhoI)	This Study
7	pBAD-His-NEDD8-Nb-G5-V5 [L110TAG]-V5	araBAD	pBR322	AMP	3,6,9,10	4	pBAD (NcoI/XhoI)	This Study
8	pBAD-His-NEDD8-Nb-G5-V5 [F112TAG]-V5	araBAD	pBR322	AMP	3,6,11,12	4	pBAD (NcoI/XhoI)	This Study
9	pBAD-His-NEDD8-Nb-G5-V5 [D113TAG]-V5	araBAD	pBR322	AMP	3,6,13,14	4	pBAD (NcoI/XhoI)	This Study
10	pBAD-His-NEDD8-Nb-G5-V5 [E116TAG]-V5	araBAD	pBR322	AMP	3,6,15,16	4	pBAD (NcoI/XhoI)	This Study
11	pBAD-His-NEDD8-Nb-G5-V5 [F117TAG]-V5	araBAD	pBR322	AMP	3,6,17,18	4	pBAD (NcoI/XhoI)	This Study
12	pBAD-14-3-3BMHI[WT]-His	araBAD	pBR322	AMP	1,2	---	pBAD (NcoI/XhoI)	This Study
13	pBAD-14-3-3-BMHI[Y133TAG]-His	araBAD	pBR322	AMP	1,2, 25, 26	12	pBAD (NcoI/XhoI)	This Study
14	pBAD-14-3-3-BMHI[Y216TAG]-His	araBAD	pBR322	AMP	1, 2, 27, 28	12	pBAD (NcoI/XhoI)	This Study
15	pBAD-14-3-3ß[WT]-His	araBAD	pBR322	AMP	1,2	---	pBAD (NcoI/XhoI)	This Study

**16**	pBAD-14-3-3ß[Y130]-His	araBAD	pBR322	AMP	1,2, 25, 26	14	pBAD (NcoI/XhoI)	This Study
**17**	pBAD-14-3-3ß[Y213]-His	araBAD	pBR322	AMP	1, 2, 27, 28	14	pBAD (NcoI/XhoI)	This Study
**18**	pBAD-AVI-14-3-3ß[WT]-His	araBAD	pBR322	AMP	1,2	---	pBAD (NcoI/XhoI)	This Study
**19**	pBAD-AVI-14-3-3ß[Y130]-His	araBAD	pBR322	AMP	1,2, 25, 26	18	pBAD (NcoI/XhoI)	This Study
**20**	pBAD-AVI-14-3-3ß[Y213]-His	araBAD	pBR322	AMP	1, 2, 27, 28	18	pBAD (NcoI/XhoI)	This Study
**21**	pEVF-GST-BirA	tac	pRSF	Chlor	19-24	---		This Study
**22**	pBAD-CaM [WT]-His	araBAD	pBR322	AMP	--	---	pBAD (NcoI/XhoI)	(Porter, Jang et al. 2020)
**23**	pBAD-CaM[99TAG]-His	araBAD	pBR322	AMP	---	---	pBAD (NcoI/XhoI)	(Porter, Jang et al. 2020)
**24**	pBAD-CaM[138TAG]-His	araBAD	pBR322	AMP	---	---	pBAD (NcoI/XhoI)	(Porter, Jang et al. 2020)
**25**	pBAD-GFP[WT]-His	araBAD	pBR322	AMP	---	---	pBAD (NcoI/XhoI)	(Miyake-Stoner, Refakis et al. 2010)
**26**	pBAD-GFP [134TAG]-His	araBAD	pBR322	AMP	---	---	pBAD (NcoI/XhoI)	(Miyake-Stoner, Refakis et al. 2010)
**27**	pBAD-GFP [150TAG]-His	araBAD	pBR322	AMP	---	---	pBAD (NcoI/XhoI)	(Miyake-Stoner, Refakis et al. 2010)
**28**	pBAD-His-SUMO-link-GFP[WT]-His	araBAD	pBR322	AMP	1, 2	25	pBAD (NcoI/XhoI)	This Study
**29**	pBAD-His-SUMO-link [TAG]-GFP [150TAG]-His	araBAD	pBR322	AMP	1, 2	27	pBAD (NcoI/XhoI)	This Study

Supplementary Table 1. – Plasmids used in the study

GENE	SEQUENCE
**His-SUMO**	AGCGCCGCCGGTGGTGAAGAAGATAAAAAACCGGCGGGCGGCGAAGGCGGTGGCGCGCATATCA<br>ACCTGAAAGTGAAAGGTCAGGATGGTAACGAAGTGTTCTTCCGCATTAAACGCAGCACCCAGCTG<br>AAGAAACTGATGAACGCCTATTGCGATCGCCAGAGCGTCGATATGACCGCCATTGCCTTCCTGTTT<br>GATGGCCGCCGTCTGCGTGCGGAACAGACGCCGGACGAACTGGAAATGGAAGACGGCGATGAAAT<br>TGATGCGATGCTGCATCAGACCGGCGG
**His-Nedd8**	ATGGGCAGCAGCCATCATCATCATCATCACAGCGGCATGATCAAAGTAAAGACCCTGACTGGCAA<br>AGAAATCGAAATCGACATCGAACCTACCGATACCATCGACCGCATCAAAGAACGTGTTGAAGAAA<br>AAGAAGGTATTCCACCGGTGCAGCAACGTCTGATCTACGCGGGTAAGCAGCTGGCGGATGATAAA<br>ACTGCGAAGGACTATAACATTGAAGGTGGCTCCGTTCTGCACCTGGTGCTGGCGCTGCGTGGTGGA<br>GGA
**AVI Tag**	GGCATGTCCGGCCTGAACGACATCTTCGAGGCTCAGAAAATCGAATGGCACGAAGGC
**GST-BirA**	ATGTCCCCTATACTAGGTTATTGGAAAATTAAGGGCCTTGTGCAACCCACTCGACTTCTTTTGGAAT<br>ATCTTGAAGAAAAATATGAAGAGCATTTGTATGAGCGCGATGAAGGTGATAAATGGCGAAACAAA<br>AAGTTTGAATTGGGTTTGGAGTTTCCCAATCTTCCTTATTATATTGATGGTGATGTTAAATTAACAC<br>AGTCTATGGCCATCATACGTTATATAGCTGACAAGCACAACATGTTGGGTGGTTGTCCAAAAGAGC<br>GTGCAGAGATTTCAATGCTTGAAGGAGCGGTTTTGGATATTAGATACGGTGTTTCGAGAATTGCAT<br>ATAGTAAAGACTTTGAAACTCTCAAAGTTGATTTTCTTAGCAAGCTACCTGAAATGCTGAAAATGT<br>TCGAAGATCGTTTATGTCATAAAACATATTTAAATGGTGATCATGTAACCCATCCTGACTTCATGTT<br>GTATGACGCTCTTGATGTTGTTTTATACATGGACCCAATGTGCCTGGATGCGTTCCCAAAATTAGTT<br>TGTTTTAAAAAACGTATTGAAGCTATCCCACAAATTGATAAGTACTTGAAATCCAGCAAGTATATA<br>GCATGGCCTTTGCAGGGCTGGCAAGCCACGTTTGGTGGTGGCGACCATCCTCCAAAATCGGATCCC<br>AAGGATAACACCGTGCCACTGAAATTGATTGCCCTGTTAGCGAACGGTGAATTTCACTCTGGCGAG<br>CAGTTGGGTGAAACGCTGGGAATGAGCCGGGCGGCTATTAATAAACACATTCAGACACTGCGTGA<br>CTGGGGCGTTGATGTCTTTACCGTTCCGGGTAAAGGATACAGCCTGCCTGAGCCTATCCAGTTACTT<br>AATGCTAAACAGATATTGGGTCAGCTGGATGGCGGTAGTGTAGCCGTGCTGCCTGTGATTGACTCC<br>ACGAATCAGTACCTTCTTGATCGTATCGGAGAGCTTAAATCGGGCGATGCTTGCATTGCAGAATAC<br>CAGCAGGCTGGCCGTGGTCGCCGGGGTCGGAAATGGTTTTCGCCTTTTGGCGCAAACTTATATTTG<br>TCGATGTTCTGGCGTCTGGAACAAGGCCCGGCGGCGGCGATTGGTTTAAGTCTGGTTATCGGTATC<br>GTGATGGCGGAAGTATTACGCAAGCTGGGTGCAGATAAAGTTCGTGTTAAATGGCCTAATGACCTC<br>TATCTGCAGGATCGCAAGCTGGCAGGCATTCTGGTGGAGCTGACTGGCAAAACTGGCGATGCGGC<br>GCAAATAGTCATTGGAGCCGGGATCAACATGGCAATGCGCCGTGTTGAAGAGAGTGTCGTTAATC<br>AGGGGTGGATCACGCTGCAGGAAGCGGGGATCAATCTCGATCGTAATACGTTGGCGGCCATGCTA<br>ATACGTGAATTACGTGCTGCGTTGGAACTCTTCGAACAAGAAGGATTGGCACCTTATCTGTCGCGC<br>TGGGAAAAGCTGGATAATTTTATTAATCGCCCAGTGAAACTTATCATTGGTGATAAAGAAATATTT<br>GGCATTTCACGCGGAATAGACAAACAGGGGGCTTTATTACTTGAGCAGGATGGAATAATAAAACC<br>CTGGATGGGCGGTGAAATATCCCTGCGTAGTGCAGAAAAATAA
**Nb-G5**	GCCATGGCCCAGGTGCAGCTGCAGGAGTCTGGGGGAGGCTTGGTGCAGCCTGGGGGGTCTCTGAG<br>ACTCTCCTGTGCAGCCTCTAAAAACATCGCCAATTTCGATTCCATGGCATGGTACCGCCGGTCTCCA<br>GGCAAGGAGCGCGACTTGGTCGCACGCCTTTATAGTGATGGCGGTACAAACTATGAAGACTCCGTG<br>AAGGGCCGATTCAACATCTCCACAGACATTGAGAAGAACACGGTTTATCTTCAAATGAACAACCTG<br>AAACTTGAGGACACGGCCCGGTATTATTGTCGCGCAGAT**CTT**GGG**TTGTTCGAT**ACATGT**GAATT**<br>**C**CCGGGCCAGGGGACCCAGGTCACCGTCTCCAGCGGCCGCTGGATCCAAAGATATCAGAGC
**14-3-3 BMHI**	ATGGgcAGCACCAGCCGTGAAGACAGCGTGTATCTGGCGAAGCTGGCGGAGCAGGCGGAACGTTAT<br>GAGGAAATGGTGGAAAACATGAAAACCGTTGCGAGCAGCGGTCAGGAGCTGAGCGTTGAGGAAC<br>GTAACCTGCTGAGCGTGGCGTACAAGAACGTTATTGGTGCGCGTCGTGCGAGCTGGCGTATCGTGA<br>GCAGCATTGAACAGAAAGAGGAAAGCAAGGAGAAAAGCGAACACCAAGTTGAGCTGATCTGCAG<br>CTATCGTAGCAAGATTGAAACCGAACTGACCAAAATCAGCGACGATATTCTGAGCGTGCTGGATA<br>GCCACCTGATTCCGAGCGCGACCACCGGCGAGAGCAAGGTTTTCTACTATAAGATGAAAGGCGAC<br>**TAC**CACCGTTATCTGGCGGAGTTTAGCAGCGGTGATGCGCGTGAAAAGGCGACCAACGCGAGCCT<br>GGAGGCGTACAAAACCGCGAGCGAGATTGCGACCACCGAACTGCCGCCGACCCACCCGATTCGTC<br>TGGGCCTGGCGCTGAACTTCAGCGTGTTTTACTATGAAATCCAGAACAGCCCGGACAAGGCGTGCC<br>ACCTGGCGAAACAAGCGTTCGACGATGCGATCGCGGAGCTGGACACCCTGAGCGAGGAAAGC**TAC**<br>AAGGATAGCACCCTGATTATGCAGCTGCTGCGTGACAACCTGACCCTGTGGACCAGCGATATGAGC<br>GAGAGCGGTCAAGCGGAAGATCAGCAACAGCAACAGCAACACCAGCAACAGCAACCGCCGGCTG<br>CGGCGGAGGGCGAAGCGCCGAAAGGTACCCATCACCATCACCATCACTGA

**14-3-3β**	ATGGGCATGGACAAATCTGAACTGGTTCAGAAAGCGAAACTGGCGGAACAGGCGGAACGTTACGA<br>CGACATGGCGGCGGCGATGAAAGCGGTTACCGAACAGGGTCACGAACTGTCTAACGAAGAACGTA<br>ACCTGCTGTCTGTTGCGTACAAAAACGTTGTTGGTGCGCGTCGTTCTTCTTGGCGTGTTATCTCTTCT<br>ATCGAACAGAAAACCGAACGTAACGAAAAAAAACAGCAGATGGGTAAAGAATACCGTGAAAAAA<br>TCGAAGCGGAACTGCAGGACATCTGCAACGACGTTCTGGAACTGCTGGACAAATACCTGATCCCG<br>AACGCGACCCAGCCGGAATCTAAAGTTTTCTACCTGAAAATGAAAGGTGACTACTTCCGT**TAC**CTG<br>TCTGAAGTTGCGTCTGGTGACAACAAACAGACCACCGTTTCTAACTCTCAGCAGGCGTACCAGGAA<br>GCGTTCGAAATCTCTAAAAAAGAAATGCAGCCGACCCACCCGATCCGTCTGGGTCTGGCGCTGAAC<br>TTCTCTGTTTTCTACTACGAAATCCTGAACTCTCCGGAAAAAGCGTGCTCTCTGGCGAAAACCGCGT<br>TCGACGAAGCGATCGCGGAACTGGACACCCTGAACGAAGAATCT**TAC**AAAGACTCTACCCTGATC<br>ATGCAGCTGCTGCGTGACAACCTGACCCTGTGGACCTCTGAAAACCAGGGTGACGAAGGT
**CaM**	ATGGCTGACCAACTTACTGAGGAACAGATTGCAGAATTCAAAGAAGCCTTCTCACTGTTTGACAAG<br>GACGGCGATGGCACGATCACTACCAAGGAACTCGGAACCGTGATGAGATCGCTCGGACAGAATCC<br>TACCGAGGCCGAACTGCAAGATATGATCAACGAAGTGGATGCGGACGGCAACGGTACCATCGACT<br>TTCCCGAATTCCTCACGATGATGGCGCGGAAGATGAAAGACACCGACAGCGAGGAAGAGATCAGG<br>GAAGCATTCCGCGTGTTCGATAAGGACGGAAACGGA**TAC**ATTTCGGCTGCGGAACTGCGCCACGT<br>GATGACTAATCTGGGGGAAAAGTTGACTGATGAAGAGGTTGACGAGATGATCCGGGAGGCCGACA<br>TCGATGGAGATGGTCAGGTCAAT**TAC**GAGGAGTTCGTCCAGATGATGACCGCCAAGGGAAAACCA<br>ATCCCGAACCCGCTGCTCGGGCTGGACTCCACTCACCATCACCATCATCACTAA
**sfGFP**	GTTAGCAAAGGTGAAGAACTGTTTACCGGCGTTGTGCCGATTCTGGTGGAACTGGATGGTGATGTG<br>AATGGCCATAAATTTAGCGTTCGTGGCGAAGGCGAAGGTGATGCGACCAACGGTAAACTGACCCT<br>GAAATTTATTTGCACCACCGGTAAACTGCCGGTTCCGTGGCCGACCCTGGTGACCACCCTGACCTA<br>TGGCGTTCAGTGCTTTAGCCGCTATCCGGATCATATGAAACGCCATGATTTCTTTAAAAGCGCGAT<br>GCCGGAAGGCTATGTGCAGGAACGTACCATTAGCTTCAAAGATGATGGCACCTATAAAACCCGTG<br>CGGAAGTTAAATTTGAAGGCGATACCCTGGTGAACCGCATTGAACTGAAAGGTATTGATTTTAAAG<br>AA**GAT**GGCAACATTCTGGGTCATAAACTGGAATATAATTTCAACAGCCAT**AAT**GTGTATATTACCG<br>CCGATAAACAGAAAAATGGCATCAAAGCGAACTTTAAAATCCGTCACAACGTGGAAGATGGTAGC<br>GTGCAGCTGGCGGATCATTATCAGCAGAATACCCCGATTGGTGATGGCCCGGTGCTGCTGCCGGAT<br>AATCATTATCTGAGCACCCAGAGCGTTCTGAGCAAAGATCCGAATGAAAAACGTGATCATATGGTG<br>CTGCTGGAATTTGTTACCGCCGCGGGCATTACCCACGGTATGGATGAACTGTATAAA
**His-SUMO-link-GFP**	ATGGGCAGCGACTCAGAAGTCAATCAAGAAGCTAAGCCAGAGGTCAAGCCAGAAGTCAAGCCTGA<br>GACTCACATCAATTTAAAGGTGTCCGATGGATCTTCAGAGATCTTCTTCAAGATCAAAAAGACCAC<br>TCCTTTAAGAAGGCTGATGGAAGCGTTCGCTAAAAGACAGGGTAAGGAAATGGACTCCTTAAGAT<br>TCTTGTACGACGGTATTAGAATCCAAGCTGATCAGACCCCTGAAGATTTGGACATGGAGGATAACG<br>ATATTATTGAGGCTCACAGAGAACAGATTGGTGGATCCGGT**TAG**GGCTCTGTTAGCAAAGGTGAA<br>GAACTGTTTACCGGCGTTGTGCCGATTCTGGTGGAACTGGATGGTGATGTGAATGGCCATAAATTT<br>AGCGTTCGTGGCGAAGGCGAAGGTGATGCGACCAACGGTAAACTGACCCTGAAATTTATTTGCACC<br>ACCGGTAAACTGCCGGTTCCGTGGCCGACCCTGGTGACCACCCTGACCTATGGCGTTCAGTGCTTT<br>AGCCGCTATCCGGATCATATGAAACGCCATGATTTCTTTAAAAGCGCGATGCCGGAAGGCTATGTG<br>CAGGAACGTACCATTAGCTTCAAAGATGATGGCACCTATAAAACCCGTGCGGAAGTTAAATTTGAA<br>GGCGATACCCTGGTGAACCGCATTGAACTGAAAGGTATTGATTTTAAAGAA**GAT**GGCAACATTCTG<br>GGTCATAAACTGGAATATAATTTCAACAGCCAT**AAT**GTGTATATTACCGCCGATAAACAGAAAAAT<br>GGCATCAAAGCGAACTTTAAAATCCGTCACAACGTGGAAGATGGTAGCGTGCAGCTGGCGGATCA<br>TTATCAGCAGAATACCCCGATTGGTGATGGCCCGGTGCTGCTGCCGGATAATCATTATCTGAGCAC<br>CCAGAGCGTTCTGAGCAAAGATCCGAATGAAAAACGTGATCATATGGTGCTGCTGGAATTTGTTAC<br>CGCCGCGGGCATTACCCACGGTATGGATGAACTGTATAAAGGCAGCCACCATCATCATCACCAT

Supplementary Table 2. – Genes used in this study. Codons that are mutated to TAG sites are indicated in red.

NO.	PRIMER	SEQUENCE
1	PBAD UNI F	CCCGTTTTTTGGGCTAACAGGAG
2	PBAD UNI R	CCCATATGGTACCAGCTGCAGATC
3	HIS-NEDD8 F	CCCGTTTTTTGGGCTAACAGGAGGAATTAACCATGGGCAGCAGCCATC ATCATCATCATCACAGCGGCATGATCAAAGTAAAGACCCTGACTGGC
4	NEDD8-PELB	GGCGCTGCGTGGTGGAGGATCACTGCTGGCAGCTCAGCCGGCCATG
5	PELB F	CACTGCTGGCAGCTCAGC
6	PBAD-V5 R	GGATCCAAAGATATCAGAGCGGCTCCGGTAAACCTATCCCAAATCCCCTGTTGG GTTTGGACTCAACTTAACTCGAGATCTGCAGCTGGTACCATATG
7	G5 [L108TAG] F	CGGTATTATTGTCGCGCAGATTAGGGGTTGTTCGATACATGTGAATTCCC
8	G5 [L108TAG] R	CACATGTATCGAACAACCCCTAATCTGCGCGACAATAATACCGGGCCGTG
9	G5 [L110TAG] F	GTCGCGCAGATCTTGGGTAGTTCGATACATGTGAATTCCCGGGCCAGG
10	G5 [L110TAG] R	GGGAATTCACATGTATCGAACTACCCAAGATCTGCGCGACAATAATACCGGGC
11	G5 [F112TAG] F	GCAGATCTTGGGTTGTAGGATACATGTGAATTCCCGGGCCAGG
12	G5 [F112TAG] R	GGAATTCACATGTATCCTACAACCCAAGATCTGCGCGACAATAATAC
13	G5 [D113TAG] F	GATCTTGGGTTGTTCTAGACATGTGAATTCCCGGGCCAGGGGACC
14	G5 [D113TAG] R	CGGGAATTCACATGTCTAGAACAACCCAAGATCTGCGCGACAATAATAC
15	G5 [E116TAG] F	GTTGTTCGATACATGTTAGTTCCCGGGCCAGGGGACCCAGGTCAC
16	G5 [E116TAG] R	CCCCTGGCCCGGGAACTAACATGTATCGAACAACCCAAGATCTGC
17	G5 [F117TAG] F	GTTCGATACATGTGAATAGCCGGGCCAGGGGACCCAGGTCACCGTC
18	G5 [F117TAG] R	GGTCCCCTGGCCCGGCTATTCACATGTATCGAACAACCCAAGATC
19	RSF F	CACTTTTCGGGGAAATGTGCTAGGCATGCAGCGCTCTTC
20	RSF R	CTAACTTACATTAATTGCGTTGCGCGGTAAACCAG
21	CMR F	TGCGTTGCGCGGTAAACCAGCAATAGACATAAG
22	CMR R	CCTCTTACGTGCCGATCAAACGCCAGCAACGCGGCCTT

23	GEX F	AACGCCAGCAACGCGGCC
24	GEX R	CCTCTGACACATGCAGCTCCCGG
25	14-3-3B [130TAG] F	GACTACTTCCGTTACCTGTCTGAAGTTGCGTCTGGTGACAACAAAC
26	14-3-3B [130TAG] R	CTACCTGAAAATGAAAGGTGACTACTTCCGTTAGCTGTCTGAAG
27	14-3-3B [213TAG] F	CTGAACGAAGAATCTTACAAAGACTCTACCCTGATCATGCAGCTG
28	14-3-3B [213TAG] R	GAACTGGACACCCTGAACGAAGAATCTtagAAAGACTCTACCCTGA
29	VHH-F	GAT GTG CAG CTG CAG GCG TCT GGR GGA GG
30	VHH-R	CGC CAT CAA GGT ACC AGT TGA

Supplementary Table 3 - Primers used in this study

References

Beyer, J. N., P. Hosseinzadeh, I. Gottfried-Lee, E. M. Van Fossen, P. Zhu, R. M. Bednar, P. A. Karplus, R. A. Mehl and R. B. Cooley (2020). "Overcoming Near-Cognate Suppression in a Release Factor 1-Deficient Host with an Improved Nitro-Tyrosine tRNA Synthetase." J Mol Biol **432**(16): 4690-4704.

Miyake-Stoner, S. J., A. M. Miller, J. T. Hammill, J. C. Peeler, K. R. Hess, R. A. Mehl and S. H. Brewer (2009). "Probing protein folding using site-specifically encoded unnatural amino acids as FRET donors with tryptophan." Biochemistry **48**(25): 5953-5962.

Miyake-Stoner, S. J., C. A. Refakis, J. T. Hammill, H. Lusic, J. L. Hazen, A. Deiters and R. A. Mehl (2010). "Generating permissive site-specific unnatural aminoacyl-tRNA synthetases." Biochemistry **49**(8): 1667-1677.

Porter, J. J., H. S. Jang, M. M. Haque, D. J. Stuehr and R. A. Mehl (2020). "Tyrosine nitration on calmodulin enhances calcium-dependent association and activation of nitric-oxide synthase." J Biol Chem **295**(8): 2203-2211.
